# Nucleotide excision repair in *Trypanosoma brucei*: specialization of transcription-coupled repair due to multigenic transcription

**DOI:** 10.1111/mmi.12589

**Published:** 2014-04-24

**Authors:** Carlos R Machado, João P Vieira-da-Rocha, Isabela Cecilia Mendes, Matheus A Rajão, Lucio Marcello, Mainá Bitar, Marcela G Drummond, Priscila Grynberg, Denise A A Oliveira, Catarina Marques, Ben Van Houten, Richard McCulloch

**Affiliations:** 1 Departamento de Bioquímica e Imunologia, ICB, Universidade Federal de Minas GeraisAv. Antônio Carlos, 6627, Caixa Postal 486, Belo Horizonte, 30161-970, MG, Brazil; 2 The Wellcome Trust Centre for Molecular Parasitology, College of Medical, Veterinary and Life Sciences, Institute of Infection, Immunity and Inflammation, University of GlasgowSir Graeme Davies Building, 120 University Place, Glasgow, G12 8TA, UK; 3 Coordenação de Pesquisa, Instituto Nacional de CâncerRua André Cavalcanti, 37 Fátima, Rio de Janeiro, 20231-050, RJ, Brazil; 4 Laboratório de Genética Animal, Escola de Veterinária, Universidade Federal de Minas GeraisAv. Antônio Carlos, 6627, Caixa Postal 486, Belo Horizonte, 30161-970, MG, Brazil; 5 Department of Pharmacology and Chemical Biology, University of Pittsburgh School of Medicine and The University of Pittsburgh Cancer Institute, Hillman Cancer CenterPittsburgh, PA, 15213, USA

## Abstract

Nucleotide excision repair (NER) is a highly conserved genome repair pathway acting on helix distorting DNA lesions. NER is divided into two subpathways: global genome NER (GG-NER), which is responsible for repair throughout genomes, and transcription-coupled NER (TC-NER), which acts on lesions that impede transcription. The extent of the *Trypanosoma brucei* genome that is transcribed is highly unusual, since most genes are organized in multigene transcription units, each transcribed from a single promoter. Given this transcription organization, we have addressed the importance of NER to *T. brucei* genome maintenance by performing RNAi against all predicted contributing factors. Our results indicate that TC-NER is the main pathway of NER repair, but only CSB, XPBz and XPG contribute. Moreover, we show that UV lesions are inefficiently repaired in *T. brucei*, perhaps due to preferential use of RNA polymerase translesion synthesis. RNAi of XPC and DDB was found to be lethal, and we show that these factors act in inter-strand cross-link repair. XPD and XPB appear only to act in transcription, not repair. This work indicates that the predominance of multigenic transcription in *T. brucei* has resulted in pronounced adaptation of NER relative to the host and may be an attractive drug target.

## Introduction

DNA genomes are exposed to a myriad of agents that modify or alter their structures. Some of these agents, like reactive oxygen species and reactive nitrogen species, can be produced endogenously, while others, such as ionizing radiation, UV light and harmful chemicals, are derived from the environment. To maintain genomic stability most living organisms possess multiple pathways to repair such DNA damage. Nucleotide excision repair (NER) is responsible for removing a remarkable variety of DNA lesions, including bulky adducts covalently attached to DNA and other forms of damage that distort the double helix structure (Naegeli and Sugasawa, 2011). NER defects in humans are associated with the cancer-prone syndrome xeroderma pigmentosum (XP) and with the developmental disorders Cockayne syndrome and tricothiodistrophy (Lehmann, 2001; Vermeulen *et al*., 2001; Emmert *et al*., 2009; Cameroni *et al*., 2010). NER is composed of two subpathways: Global Genome Nucleotide Excision Repair (GG-NER), which recognizes lesions genome-wide, and Transcription-Coupled NER (TC-NER), which recognizes DNA lesions that block elongation of RNA polymerase (Pol) in the transcribed strand of active genes (Naegeli and Sugasawa, 2011). It is widely considered that GG-NER and TC-NER operate together in all organisms to maintain genome fidelity. In this study we have asked whether or not such parallel functioning of the two NER pathways is maintained in trypanosomatids, eukaryotes in which gene expression is highly diverged.

During GG-NER in humans XPC (XP factor C), together with RAD23b and Centrin-2 (Mu *et al*., 1995; Araujo *et al*., 2000; Araki *et al*., 2001; Nishi *et al*., 2005), recognizes local strand openings associated with double strand helix-distorting lesions (Min and Pavletich, 2007). In some cases, XPC recruitment can be aided by a dimer of the UV-damaged DNA binding (UV-DDB) protein, composed of DDB1 and XPE (DDB2) subunits, the latter of which recognizes UV-induced lesions in the context of chromatin (Feldberg and Grossman, 1976; Chu and Chang, 1988). The damage is then handed off to XPC, which promotes loading of the TFIIH transcription factor complex through the NER protein’s C-terminal domain. TFIIH is composed of 10 proteins, divided into two subcomplexes: the core, which contains two helicases, XPB and XPD, and five associated proteins (p62, p52, p44, p34 and p8/TTD-A); and CAK, the kinase activating complex (Compe and Egly, 2012). TFIIH is recruited by XPC interaction with XPB and with p62, allowing damage excision to begin by opening the DNA helix around the lesion. The XPB and XPD helicases act in this reaction, though it appears that XPD helicase activity is crucial, while XPB provides ATPase, but not helicase, activity (Oksenych and Coin, 2009). Once DNA denaturation is established, RPA is loaded to protect the single-stranded DNA in the bubble. At the same time XPA is also loaded in order to stabilize the TFIIH-XPC complex, as well as to cause release of the CAK subcomplex, which coincides with recruitment of the NER incision endonucleases, XPF/ERCC1 and XPG. XPF/ERCC1 catalyses incision 5′ of the lesion, while XPG executes an incision 3′. This leads to excision of a DNA oligonucleotide containing the lesion, though the 3′ incision appears to occur after Replication Factor C and Proliferating Cell Nuclear Antigen are recruited and co-ordinate loading of the replication machinery needed for repair, composed of DNA Pols delta and kappa in non-dividing cells, and DNA Pol epsilon in replicative cells (Ogi *et al*., 2010). Following gap-filling, the remaining nick is sealed by DNA ligase I or DNA ligase III in dividing and non-dividing cells respectively (Fagbemi *et al*., 2011).

TC-NER differs from GG-NER in the DNA damage recognition step, which is made through identification of stalled RNA Pol at lesions in transcribed genes. RNA Pol II (and perhaps also RNA Pol I) blockage increases interaction of the polymerase with the Cockayne syndrome type B (CSB) protein, an SNF2 ATPase, which may stimulate RNA Pol translocation along the DNA strand, allowing TFIIH complex access to the lesion site (Hanawalt and Spivak, 2008). From then, the TC-NER and GG-NER pathways proceed in the same way. In TC-NER ongoing transcription can be aborted if RNA Pol disengages with the template, or may resume if the enzyme complex remains attached, which may require the action of CSA in eukaryotes, perhaps by antagonizing CSB (Hanawalt and Spivak, 2008). XPG has also been shown to interact with CSB and RNA Pol II, and may be responsible for allowing NER to occur without release of RNA Pol (Sarker *et al*., 2005). NER may not occur in all cases, however. In some circumstances, RNA Pol can insert nucleotides opposite lesions, including UV damage (Walmacq *et al*., 2012), and extend beyond the blockage in an error-prone fashion, a process termed transcriptional mutagenesis (Saxowsky and Doetsch, 2006; Bregeon and Doetsch, 2011). Though such translesion transcription through damaged DNA templates can lead to mutant transcripts, this process rescues cells from death by severe stalling of RNA Pol, a strong signal for apoptosis.

Trypanosomatids are flagellated protists, which may have emerged early during eukaryotic evolution and display several highly diverged biological processes. *Trypanosoma cruzi*, *Trypanosoma brucei* and *Leishmania sp* are among the most studied organisms of this family and are sources, respectively, of Chagas disease in Latin America, sleeping sickness in sub-Saharan Africa and leishmaniasis in tropical areas, collectively affecting more than 20 million people (http://www.who.int/topics/en/, last accessed 2012). To date, only a few studies have examined NER in these organisms. The machinery is largely conserved (see *Results*), though genome sequencing failed to reveal orthologues of the smaller subunit of the UV-DDB dimer (DDB2), XPA or CSA (Berriman *et al*., 2005; El-Sayed *et al*., 2005; Ivens *et al*., 2005), and two distinct XPB-like proteins were identified, designated TbXPB and TbXPBz (Lecordier *et al*., 2007; Badjatia *et al*., 2013). Furthermore, trypanosomatid TFIIH lacks the CAK complex and associates with two trypanosomatid-specific proteins, TSP1 and TSP2 (Lee *et al*., 2009). The implications for NER of these changes are unknown; for instance, CAK disassociation from TFIIH is considered a critical step for recruitment of XPF/ERCC1 and XPG to the lesion site during NER (Svejstrup *et al*., 1995; Coin *et al*., 2008). More broadly, the genomes of trypanosomatids provide a unique eukaryotic landscape in which NER must operate. Here, the vast majority of protein-coding genes are found in directional gene clusters, each of which is transcribed from a single promoter, generating primary transcripts from which individual mRNAs are derived by trans-splicing (Daniels *et al*., 2010). Thus, trypanosomatid chromosomes contain few promoters (Siegel *et al*., 2009), which may be constitutively active, meaning that the extent of the genome that is transcribed and the uniform level of this transcription is unprecedented in eukaryotes, where one gene/one promoter and variable gene transcription rates are considered the norm. The consequences of this genetic organization for trypanosomatid genome stability have been little explored. For instance, collisions between the DNA replisome and RNA Pol at genes transcribed during S phase are associated with genomic instability, which is limited by the evolution of replication and transcription co-directionality in some highly expressed genes in bacteria and humans (Kim and Jinks-Robertson, 2012). Remarkably, replication origin mapping in *T. brucei* has shown that multigenic transcription units can meet replication forks head-on and slow their progress, despite the prediction that such collisions would have a pronounced cost in this genome (Tiengwe *et al*., 2012).

**Table 1:** Putative NER factors in *T. brucei* (including gene ID from http://tritrypdb.org), and the phenotypes detected after RNAi

Factor name	Gene ID	Growth impairment?	UV sensitive?	Cisplatin sensitive?	Cyclophosphamide sensitive?
TbXPC	Tb927.9.11930	+	−	−	+
TbRAD23	Tb927.6.4650	N/D	N/D	N/D	N/D
TbDDB	Tb927.6.5110	+	−	−	+
TbCSB	Tb927.7.4080	(−)	+	+	−
TbXPBz	Tb927.11.16270	−	+	+	−
TbXPB	Tb927.3.5100	+	−	−	−
TbXPD	Tb927.8.5980	+	−	−	−
TbXPG	Tb927.9.11760	−	−	+	−
TbERCC1	Tb927.7.2060	N/D	N/D	N/D	N/D
TbXPF	Tb927.5.3670	N/D	N/D	N/D	N/D

‘+’ indicates effect; ‘−’ indicates no effect; ‘(−)’ indicates weak effect; and ‘N/D’ indicates not determined.

In this study, we have examined how trypanosomatid gene organization influences NER. *A priori*, it might be predicted that the transit of RNA Pol through promoter-distal genes of a multigene transcription unit encounters a greater number of lesions than promoter-proximal genes. Indeed, UV treatment provided the first evidence for multigene transcription in *T. brucei*, causing greater impediment to the transcription in cell extracts of a Variant Surface Glycoprotein (VSG) gene ∼ 60 kbp from its RNA Pol I promoter than Expression Site Associated Genes found within the same transcription unit but closer to the promoter (Johnson *et al*., 1987; Coquelet *et al*., 1989). The same differential UV effect is seen for genes residing in RNA Pol II multigene transcription units (Martinez-Calvillo *et al*., 2003;2004). Despite these observations, there is no evidence that mRNA levels show a simple inverse correlation with distance of genes from the promoter (Poon *et al*., 2012). If, and how, NER in these parasites might contribute to the maintenance of gene expression and function in the context of multigenic transcription has not been examined. To investigate this question, we have performed RNAi against all putative annotated NER factors in bloodstream form (BSF; mammal-derived) *T. brucei*, both in the absence and presence of genotoxic agents, and have analysed relative survival and mRNA abundance across an RNA Pol II polycistron. We have also evaluated DNA repair kinetics in *T. brucei* and *T. cruzi* cells exposed to UV irradiation and cisplatin. We show that TbXPC, TbDDB, TbXPB and TbXPD are essential in *T. brucei*, but we find no evidence that these factors act in NER. Our data show that TC-NER is the major NER pathway in this organism, though only TbCSB, TbXPG and TbXPBz act in this reaction, at least among the genes examined. In addition, we suggest that translesion RNA synthesis is favoured over repair and this pathway selection is mediated by CSB. Taken together, these results suggest that genome predictions of parallel functioning of TC-NER and GG-NER are incorrect and instead there is pronounced separation of NER pathways in *T. brucei*, an evolutionary adaptation that is associated with specialization of the NER machinery.

## Results

### Growth of bloodstream form *T. brucei* following RNAi knockdown of putative NER components

Genome sequencing of *T. brucei* revealed the presence of most NER factors, suggesting that GG-NER and TC-NER pathways are present in this parasite and in *T. cruzi* and *L. major* (Berriman *et al*., 2005; El-Sayed *et al*., 2005; Ivens *et al*., 2005). Sequence comparisons of the predicted *T. brucei* NER proteins relative to their counterparts found in other organisms were performed (Figs S1–S9), and gene IDs are provided in Table 1. Among GG-NER factors, a *T. brucei* orthologue of XPC (TbXPC) and its binding partner RAD23 were clearly conserved, and a putative orthologue of the larger of the UV-DDB dimer subunits showed weak homology to DDB1 proteins from *Homo sapiens* and *Arabidopsis thaliana*. The smaller DDB2 subunit could not be found in *T. brucei* and for simplicity we refer to the conserved *T. brucei* subunit as TbDDB. For TC-NER, a CSB orthologue was readily identified, but no clear CSA-encoding gene. Sequence analysis of TbXPB, TbXPBz and TbXPD made previously (Lecordier *et al*., 2007; Badjatia *et al*., 2013) was confirmed (data not shown). The presence of two XPB-like proteins is not unique to trypanosomatids, but is also seen in plants (Morgante *et al*., 2005) and in most crenarchaea (Richards *et al*., 2008). However, the trypanosomatid proteins, like those in the archaea, display considerable sequence and size divergence, whereas the two plant XPBs are very closely related paralogues (Fig. S6). Thus, functional divergence of the two XPB factors is more likely in trypanosomatids and in archaea than in plants. Finally, the predicted NER endonucleases could be identified. TbXPG is substantially smaller (746 amino acid residues) than *H. sapiens* and *A. thaliana* orthologues (both greater than 1100 amino acids; Fig. S7), due to the absence of residues in the parasite protein within the human XPG ‘spacer’ region that is important in TFIIH interaction and separates conserved N- and I-region nuclease domains (Dunand-Sauthier *et al*., 2005). Putative orthologues of both subunits of the XPF/ERCC1 complex could be identified, though TbXPF was more clearly conserved than TbERCC1.

**Figure 1:**
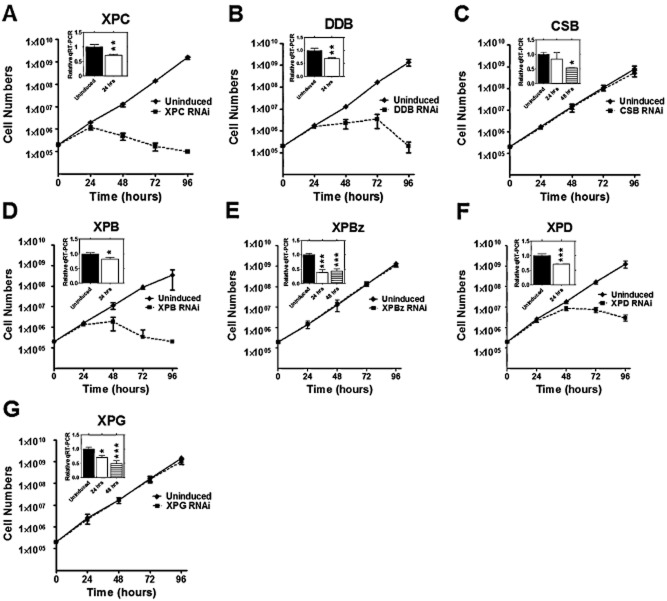
Growth curves of *T. brucei* cells following RNAi of NER factors. Growth curves are shown of *T. brucei* bloodstream form cells in the absence (solid lines) or presence (dotted lines) of tetracycline induction of RNAi against the putative NER factors TbXPC, TbDDB, TbCSB, TbXPB, TbXPBz, TbXPD and TbXPG (A–G respectively). Cell concentrations were multiplied by dilutions made during growth to obtain the cumulative amount of parasites during the time-course. Data points are the mean of three experiments, each performed in triplicate, and vertical lines denote standard deviation. In each growth curve, the insert shows qRT-PCR quantification of the levels of mRNA for the RNAi-targeted gene 24 (white box) or 48 h (hatched) after induction of RNAi, relative to mRNA levels before induction (black); vertical lines denote standard deviation from three experiments and *, ** and *** indicate *P*-values of < 0.05, < 0.01 and < 0.001 respectively.

Constructs for the generation of stem-loop RNAs against all the above ORFs except *TbRAD23* were produced and introduced into BSF *T. brucei* cells that allow the tetracycline-controlled induction of RNAi (Alsford and Horn, 2008; Jones *et al*., 2014). To test for the induction of RNAi, mRNA levels of each target NER gene from the respective cell line were measured by quantitative RT-PCR in the presence and absence of tetracycline; significant loss of mRNA was detectable for most genes 24–48 h post RNAi induction (Fig. 1). However, RNAi of TbXPF or TbERCC1, though resulting in pronounced cell growth and DNA damage response phenotypes, unexpectedly caused increased, not decreased, levels of mRNA, a response that we cannot yet explain (data not shown). For this reason, the effects of RNAi on these two putative NER genes are not detailed here. Cell growth was measured to determine the effect of the RNAi inductions (Fig. 1), which revealed that TbXPC, TbDDB, TbXPB and TbXPD are essential in this life cycle stage, since pronounced growth impairment was seen following RNAi of each gene product (Fig. 1A, B, D and F). In all cases, growth reduction was seen 24–48 h post RNAi induction, and this was followed in 24–48 h by cell death. The growth profile we observed following RNAi of TbXPB and TbXPD closely matched that described previously (Lecordier *et al*., 2007). In contrast, RNAi of further putative *T. brucei* NER components had much less impact. RNAi of TbCSB resulted in a slightly reduced growth rate compared with non-induced controls, though this was only detectable beyond 4 days after RNAi induction (Fig. 1C and data not shown). RNAi against TbXPBz and TbXPG had no discernible effect on cell growth (Fig. 1E and G), even up to 7 days post induction (data not shown); for TbXPBz, this finding is consistent with observations by Badjatia *et al*. (2013).

### TbCSB and TbXPBz are involved in DNA damage recovery after UV irradiation

**Figure 2:**
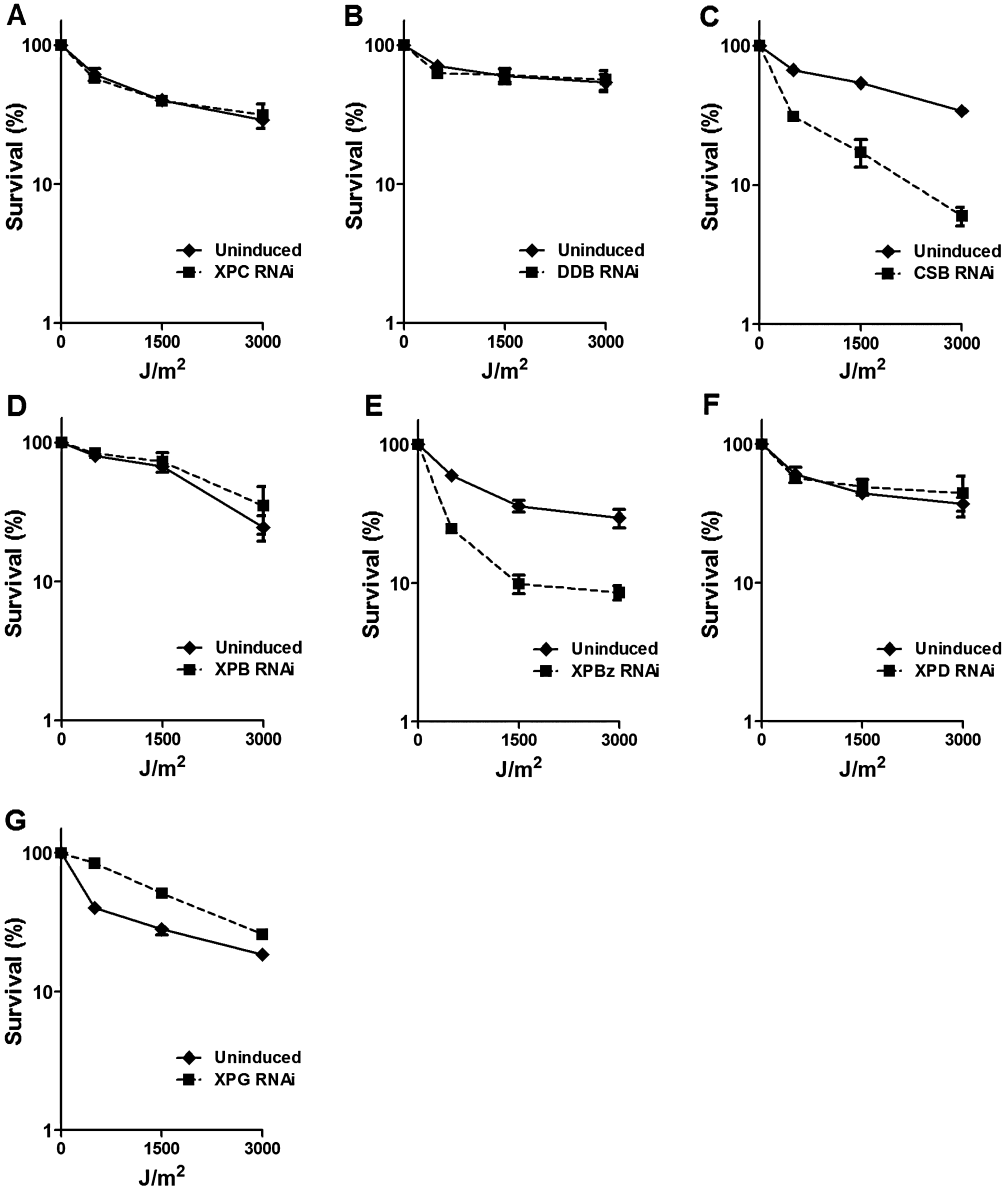
TbCSB and TbXPBz are involved in DNA damage recovery after UV irradiation. Survival curves of *T. brucei* bloodstream form cells after treatment with 500, 1500 or 3000 J m^−2^ of UVC in the absence (solid lines) or presence (dotted lines) of tetracycline induction of RNAi against NER factors TbXPC, TbDDB, TbCSB, TbXPB, TbXPBz, TbXPD and TbXPD (A–G respectively). Percent survival was calculated as the relative number of cells in the treated groups compared with untreated controls. Values are the means of three experiments, and vertical lines denote standard deviation.

Ultraviolet radiation causes cyclobutane pyrimidine dimers (CPDs) and 6-4 photoproduct (6-4 PP) lesions in DNA, which can be repaired either by direct repair in organisms endowed with photolyase (Beukers *et al*., 2008) or by NER. To determine the role of the putative *T. brucei* NER genes in DNA damage recovery after UV irradiation, growth of the RNAi cells was measured after treatment with UVC. To do this, 24 h after the addition of tetracycline to induce RNAi, the cells were exposed to 0, 500, 1500 or 3000 J m^−2^ UVC and cell density measured 24 h later; control cells in which RNAi was not induced were treated in the same way. Survival of the cells was determined by calculating the cell density of the RNAi-induced or uninduced cells at the different levels of UVC relative to untreated cells (Fig. 2). TbCSB and TbXPBz were found to play an important function in *T. brucei* survival after UV exposure, since RNAi against these two factors resulted in a pronounced increase in cell death relative to non-RNAi induced controls. In contrast, RNAi of TbXPG led to somewhat increased survival, suggesting that the endonuclease antagonizes UV damage processing, at least in these conditions. Finally, RNAi of TbXPC, TbDDB, TbXPB or TbXPD had no effect on survival after UV irradiation, suggesting that UV lesions in *T. brucei* are not recognized by the putative GG-NER proteins TbXPC and TbDDB, and nor are they acted upon by the parasite TFIIH-associated helicases.

### UV-induced lesions are not always repaired in trypanosomatids

**Figure 3:**
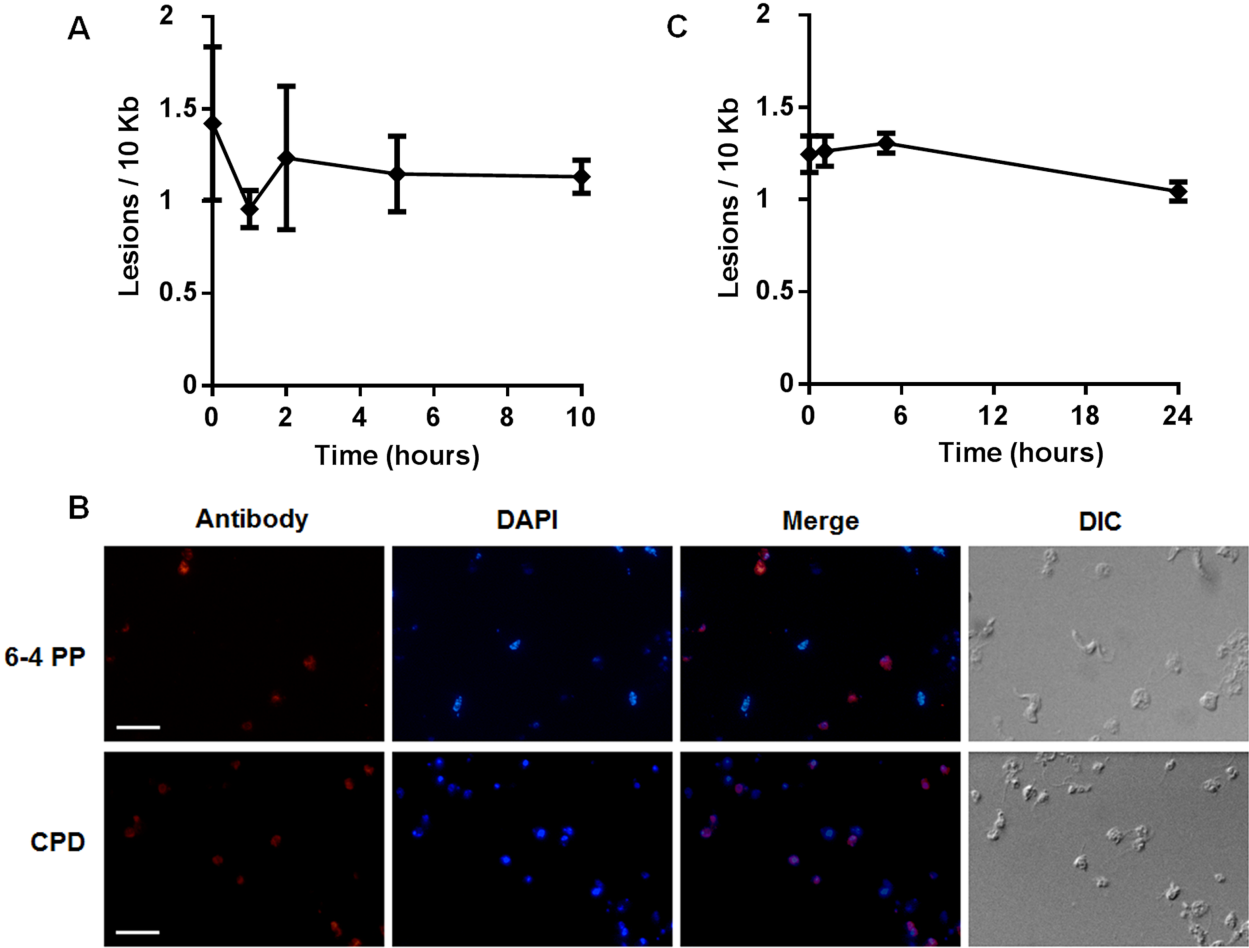
UV-induced lesions are not always repaired in *T. brucei*.A. Number of lesions per 10 kb in the nucleus of bloodstream form *T. brucei* versus time in hours, after 1500 J m^−2^ of UVC irradiation. The concentration of DNA lesions was measured up to 10 h after DNA damage induction. Each value is the mean of two biological replicates, and were derived from relative quantitative PCR-amplification values obtained from two independent measurements.B. Immunofluorescence microscopy of 6-4 photoproducts (6-4 PP; upper) and cyclobutane pyrimidine dimers (CPD; lower) in bloodstream form *T. brucei* after treatment with 1500 J m^−2^ of UVC. The scale bar represents a length of 10 μM.C. Number of lesions per 10 kb in the nucleus of epimastigote from *T. cruzi* versus time in hours, after exposure to 1500 J m^−2^ of UVC. The concentration of DNA lesions was measured up to 24 h after treatment, as above.

The above observations could be explained by UV-induced lesions being repaired predominantly by TC-NER, mediated by TbCSB and TbXPBz. Alternatively, UV damage may in some circumstances be bypassed, such as by translesion RNA synthesis, a hitherto unseen pathway in trypanosomatids. In order to address this, the kinetics of UV-induced DNA damage repair were analysed using a quantitative, PCR-based technique (Santos *et al*., 2006). Cells were treated with 1500 J m^−2^ of UV radiation and lesion density within a ∼ 10 kbp region of transcribed nuclear DNA measured at a number of time points after exposure. At this level of UV exposure, ∼ 1.5 PCR-blocking lesions were found in the 10 kbp locus tested, and repair was remarkably slow, since there was no clear reduction in lesion density for up to 10 h post treatment (Fig. 3A). The DNA repair kinetic analysis was performed only within this time frame as it corresponds with the approximate doubling time of BSF *T. brucei* cells, thus minimizing the effect of newly synthesized DNA, which would dilute the damaged templates. To address if DNA damage caused by UV radiation persists beyond a single cell cycle, antiserum against CPDs and 6-4 PPs were used to detect these lesions by immunofluorescence, and signal was readily detected 24 h after UV treatment (Fig. 3B). To determine if these slow repair kinetics are limited to *T. brucei*, the PCR assay was performed in epimastigote *T. cruzi* cells. PCR-blocking lesions accumulated to a very similar extent, and there was little evidence for repair of the UV-induced lesions for up to 24 h post treatment (Fig. 3C).

### TbCSB, TbXPBz and TbXPG participate in TC-NER of lesions induced by cisplatin

**Figure 4:**
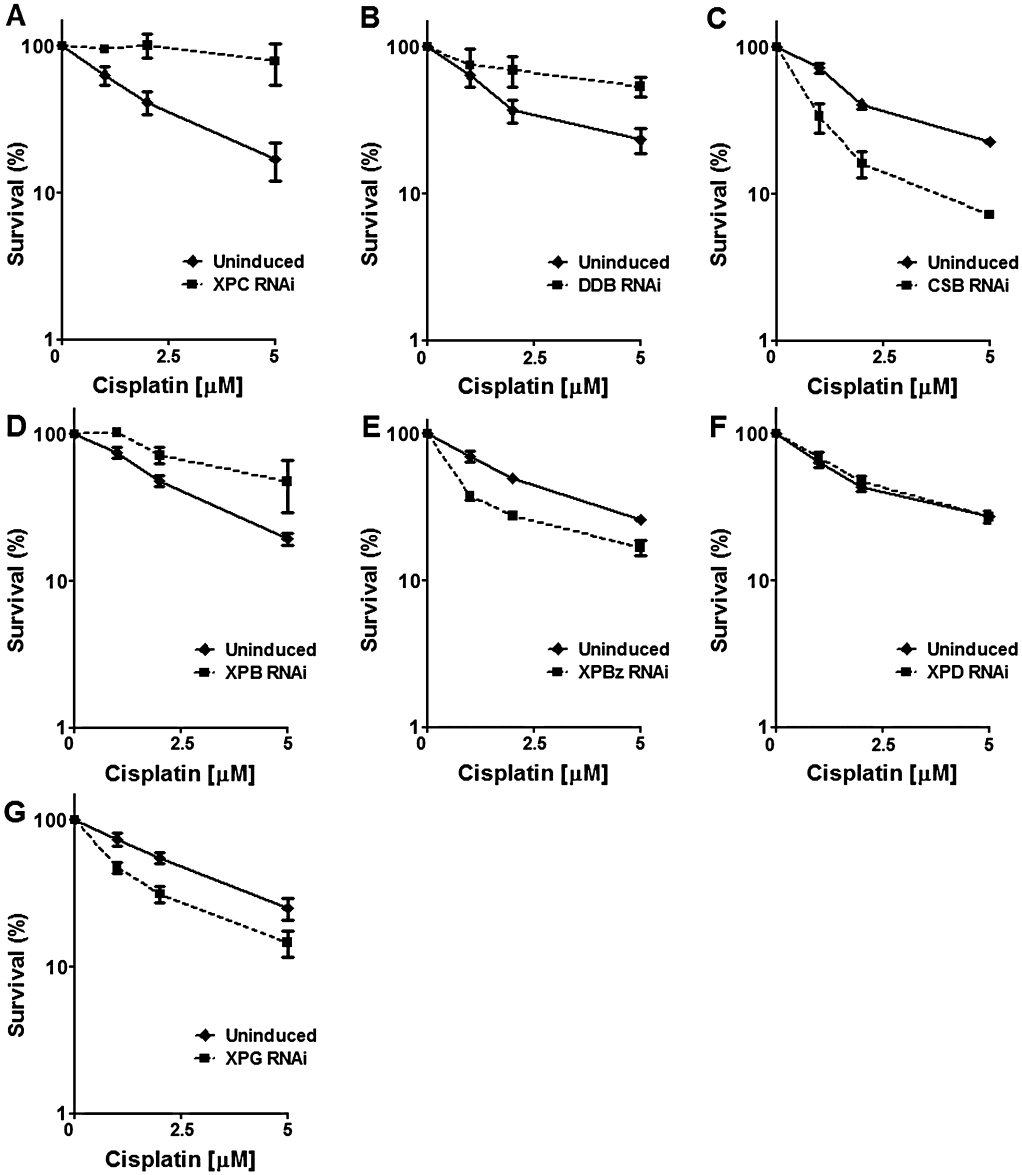
TbCSB, TbXPBz and TbXPG participate in TC-NER of lesions induced by cisplatin. Survival curves of TbXPC, TbDDB, TbCSB, TbXPB, TbXPBz, TbXPD and TbXPD (A–G respectively) RNAi depleted cells (dotted lines) compared with respective uninduced controls (solid lines) after exposure to 1, 2 or 5 μM cisplatin. Percent survival was calculated as the relative number of cells present in the treated groups compared with untreated control. Values are means of three experiments, and vertical lines denote standard deviation.

Cisplatin has been used for over 30 years in the treatment of many types of cancer (Stordal and Davey, 2007). It induces predominantly intrastrand cross-links between adenine and guanine residues and between adjacent guanines; a minor proportion of cisplatin damage is interstrand cross-links (Masters and Koberle, 2003). Cisplatin-induced intrastrand cross-links are mainly repaired by NER. To investigate the role of NER in responding to cisplatin in *T. brucei*, the BSF RNAi cell lines were cultivated in the presence of 0, 1, 2 or 5 μM cisplatin, with and without RNAi induction, and survival measured as before (Fig. 4). RNAi knockdown of TbCSB or TbXPBz resulted in increased sensitivity to cisplatin, similar to that seen for UV. In addition, RNAi of TbXPG, which did not appear to act in the response to UV, also rendered the parasites sensitive to cisplatin. These data indicate that TC-NER facilitates repair of cisplatin DNA damage, recruiting the XPG endonuclease for this purpose. In contrast, RNAi against TbXPC, TbDDB or TbXPB resulted in increased tolerance to cisplatin, perhaps suggesting that these putative GG-NER factors can recognize adducts induced by cisplatin, but this does not direct the lesions to repair. RNAi of TbXPD did not affect cisplatin sensitivity.

### Lesions generated by cisplatin are removed from trypanosomatid DNA with high efficiency by TC-NER

**Figure 5:**
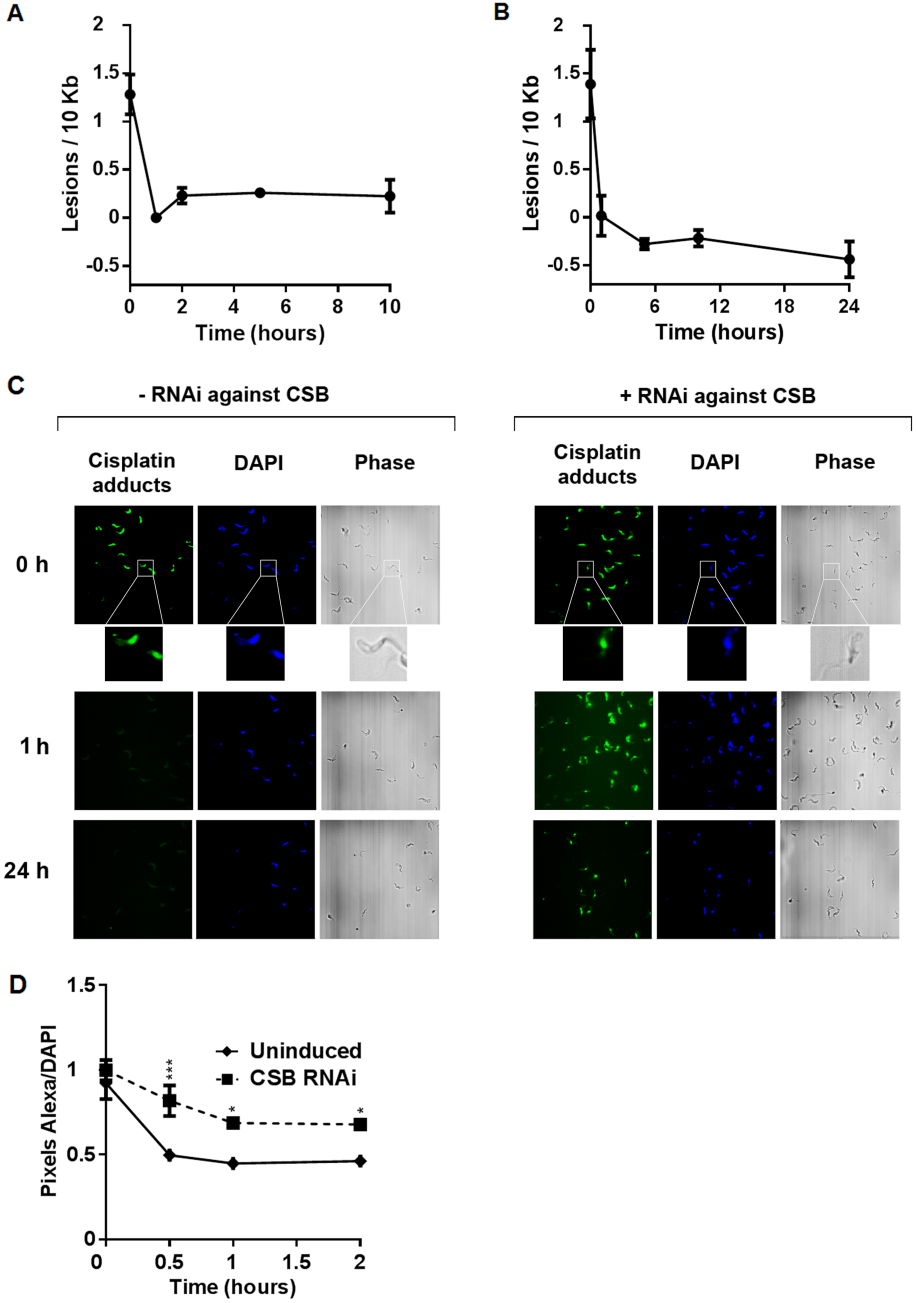
Lesions generated by cisplatin are removed from trypanosomatid DNA with high efficiency.A. Number of lesions per 10 kb in the nucleus of *T. brucei* bloodstream form cells versus time in hours after the treatment with 100 μM of cisplatin for 1 h. The concentration of DNA lesions was measured up to 10 h after the treatment. Each value is the mean of two biological replicates, and were derived from relative quantitative PCR amplification values obtained from two independent measurements.B. Number of lesions per 10 kb in the nucleus of *T. cruzi* epimastigote forms versus time in hours, after the treatment with 300 μM cisplatin for 1 h. The concentration of DNA lesions was measured up to 24 h after the treatment, as above.C. Immunofluorescence microscopy of cisplatin lesions (cisplatin adducts) in BSF *T. brucei* cells one hour after exposure to 50 μM cisplatin, with (+ RNAi) or without (− RNAi) prior induction of RNAi against TbCSB for 48 h (0 h; inserts provide a focus on individual cells to show the predominantly nuclear cisplatin signal relative to the DAPI stained and phase contrast images). Below are images of the RNAi induced or uninduced cells, 1 and 24 h after exposure to cisplatin.D. Quantification of cisplatin immunofluorescence relative to DAPI in individual TbCSB RNAi-induced cells (dotted line) and uninduced controls (solid line) 0, 1 and 24 h after exposure to 50 μM cisplatin. Values are mean pixels ratios (Alexa/DAPI), measured from all parasite nuclei present in ten different fields of each cell and at each time point; bars depict standard deviation, and * and *** indicate *P*-values of < 0.05 and < 0.001 respectively.

To determine the efficiency of removal of lesions generated by cisplatin, the kinetics of DNA repair were measured by the quantitative PCR assay after *T. brucei* BSF cells had been exposed to 100 μM cisplatin for one hour. In these conditions ∼ 1.5 lesions were formed in the 10 kbp region analysed and DNA repair was highly efficient, since virtually all lesions were removed after one hour (Fig. 5A). The DNA repair efficiency was also measured in epimastigote *T. cruzi* cells, revealing the same rapid repair (Fig. 5B). These data indicate that DNA injury caused by cisplatin is processed differently from UV. To test if the removal of cisplatin is mediated by TC-NER, we performed immunofluorescence with antiserum against cisplatin (Fig. 5C and D). TbCSB RNAi cells were exposed to 50 μM cisplatin for one hour, with or without prior induction of RNAi for 24 h, and the cells were visualized at this time point and at one and 24 h subsequently. With or without RNAi, cisplatin damage was detectable in the nucleus of the cells immediately after treatment. In the absence of RNAi the nuclear signal was no longer detectable one and 24 h later, consistent with the rapid repair indicated by the quantitative PCR assay. In contrast, a pronounced nuclear signal for cisplatin was seen in the TbCSB RNAi-induced cells one and 24 h after exposure. Moreover, quantification of the cisplatin signal in individual nuclei confirmed increased persistence of cisplatin lesions in individual cells after RNAi of TbCSB. These data indicate that cisplatin repair is impeded by the loss of TbCSB.

**Figure 6:**
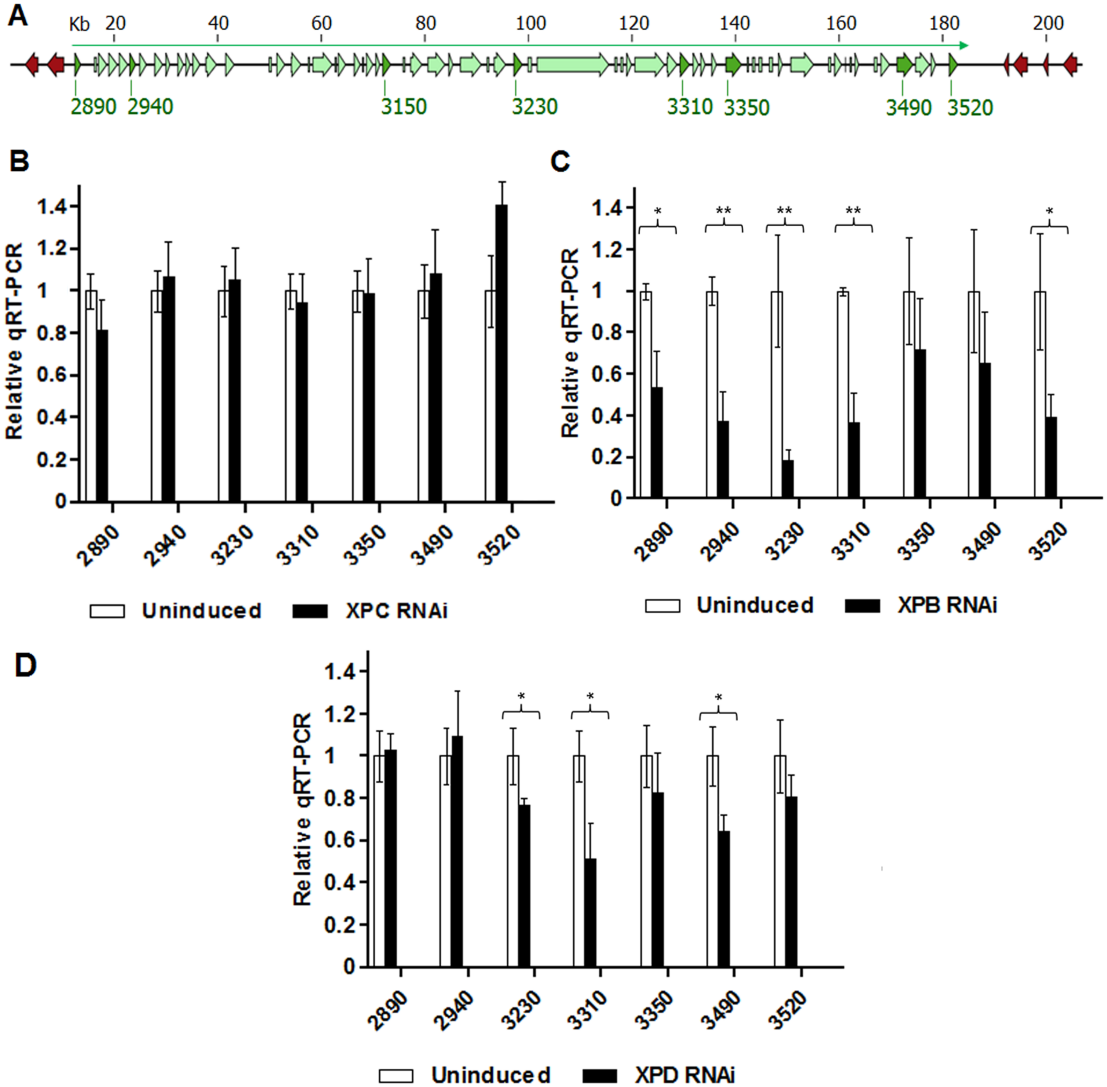
Evaluation of mRNA levels following RNAi of NER factors in *T. brucei*. (A) A region of Lister 427 *T. brucei* chromosome 8, spanning nucleotides 860 000–1 072 999, is shown. Within this region a ∼ 170 kbp multigenic transcription unit spanning 63 genes (green boxed arrows) is indicated (green arrow above the genes denotes the extent of transcription in a left to right direction in this region), as well as some genes from adjacent transcription units (red arrows; transcription not shown). Direction of transcription and translation is indicated by the arrows, and the size of the locus in shown (kb). mRNA levels were measured for seven genes, which are highlighted in dark green and labelled to reflect gene nomenclature in http://tritrypdb.org: 2890 (Tb427.08.2890), 2940 (Tb427.08.2940), 3230 (Tb427.08.3230), 3310 (Tb427.08.3310), 3350 (Tb427.08.3350), 3490 (Tb427.08.3490) and 3520 (Tb427.08.3520). mRNA levels were compared by qRT-PCR in the absence or presence of tetracyclin induction of RNAi against the putative NER factors TbXPC (B), TbXPB (C) or TbXPD (D). * and ** indicate *P*-values < 0.05 and < 0.01 respectively.

### Evaluation of mRNA levels following RNAi of NER factors in *T. brucei*

**Figure 7:**
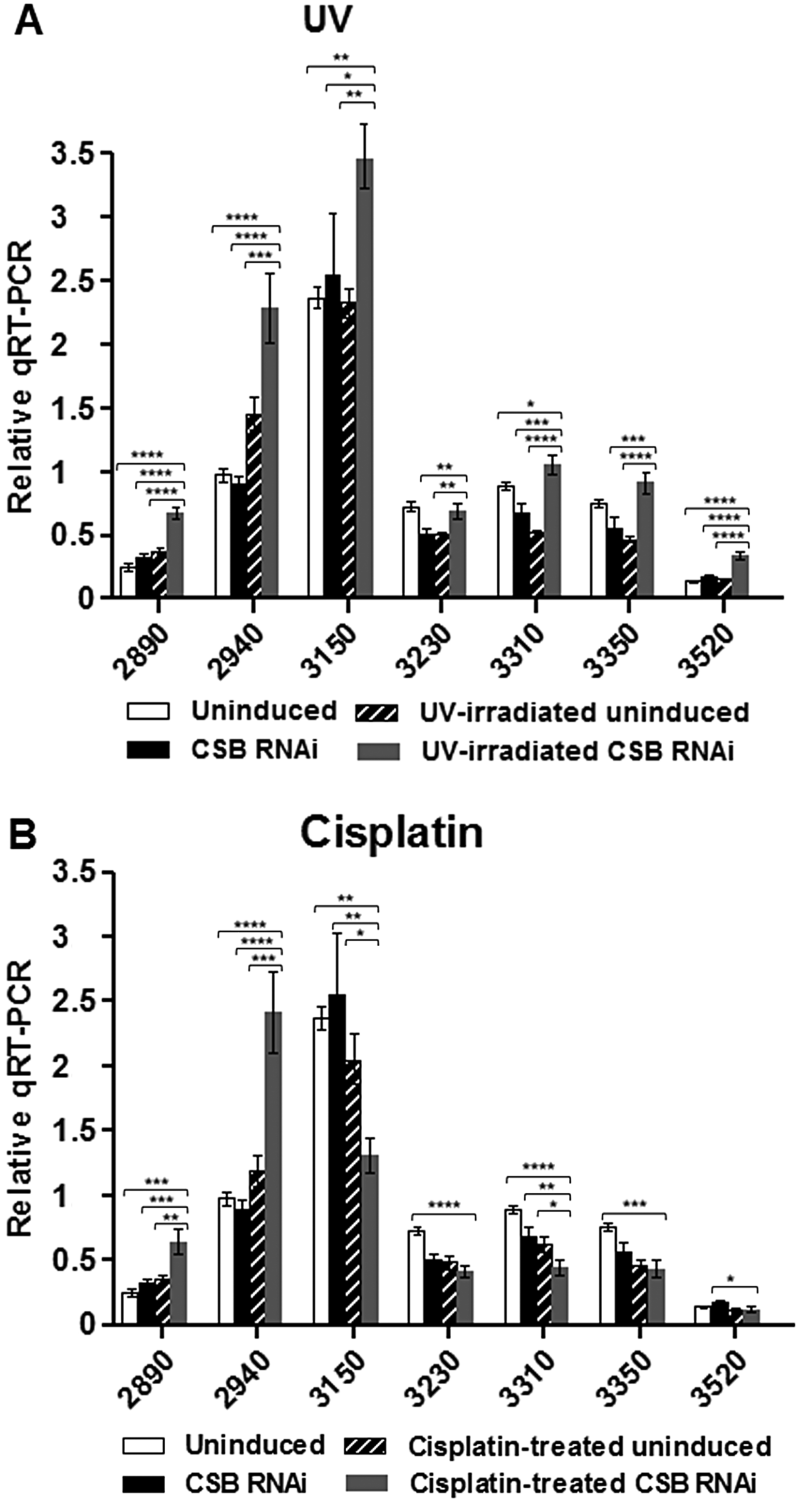
Evaluation of mRNA levels following TbCSB RNAi and after DNA damage. The mRNA levels of genes 2890, 2940, 3150 (T7.08.3150), 3230, 3310, 3350 and 3520 (see Fig. 6) are shown in RNAi-induced cells, with and without DNA damage, compared with non-induced controls. Statistical analyses comparing only the RNAi plus DNA damage groups (grey bars) against respective controls (white, black and cross-hatched bars) are represented: *, **, *** and **** indicate *P*-values of < 0.05, < 0.01, < 0.001 and < 0.0001 respectively.A. DNA damage induced by 1500 J m^−2^ UVC.B. DNA damage induced after exposure to 50 μM cisplatin for 3 h.

To investigate the impact that RNAi against NER factors has on transcription, mRNA levels of a number of genes spanning an ∼ 170 kbp multigenic transcription unit (localized around the region used in the assay of DNA repair kinetics) were measured by qRT-PCR before and after RNAi induction (Fig. 6). RNAi against four of the seven putative NER genes had no strong effect on the abundance of the transcripts examined (mRNA levels are shown in Fig. 6B for TbXPC as an example; TbDDB, TbXPBz and TbXPG, data not shown). The effect of TbCSB RNAi is discussed below (Fig. 7). RNAi of TbXPB or TbXPD caused a decrease in mRNA levels: RNAi of the former significantly reduced mRNA levels for five of the seven analysed genes, while RNAi of the later significantly reduced mRNA levels for three of the seven genes (Fig. 6C and D). This most likely reflects the role of TbXPB and TbXPD as constituents of the TbTFIIH complex *in vivo*, and is consistent with TbXPD contributing to SL-RNA transcription (Lee *et al*., 2009).

### Evaluation of mRNA levels following TbCSB RNAi and after DNA damage

To assess the role of TbCSB in transcription after DNA damage, mRNA levels of genes in the polycistron were next measured following RNAi of TbCSB and exposure to UV irradiation or cisplatin (Fig. 7). In the absence of DNA damage RNAi of TbCSB reduced the mRNA abundance of three of the four genes most distal from the transcription start (3230, 3310, 3350). Treatment with UV, in the absence of TbCSB RNAi, caused the same effect in these three transcripts, and additionally led to small increases in mRNA levels of the two genes most proximal to the transcription start (within ∼ 10 kbp; 2890 and 2940). Taken together, these data show that UV damage or TbCSB RNAi affects mRNA abundance, but in complex ways, possibly dependent on location within a polycistron. When TbCSB was depleted by RNAi and the cells were exposed to UV, mRNA levels increased significantly for five of the seven genes analysed when compared with the untreated and uninduced control. In addition, increased mRNA levels were seen for all genes relative to the TbCSB RNAi-induced, non-UV treated and to the non-RNAi-induced, UV-irradiated cells. All these effects were most pronounced for the three genes most proximal (∼ 2, 10 and 60 kbp) to the transcription start site (Fig. 7A). These data suggest that loss of TbCSB increases the generation or stability of mRNAs in the presence of UV damage.

Distinct effects to those seen with UV were observed after exposure to cisplatin (Fig. 7B). As was seen following UV, mRNA levels of the two genes most proximal to the promoter (2890 and 2940) increased after TbCSB RNAi induction and drug exposure relative to all other conditions, and again the mRNA levels for genes 3230, 3310, 3350 reduced after TbCSB RNAi or cisplatin exposure. However, unlike after UV treatment, mRNAs from the five genes situated further downstream in the polycistron decreased after TbCSB RNAi and cisplatin exposure relative to the levels seen in the uninduced and untreated control (Fig. 7B). These data suggest that TbCSB mediates a different NER response to damage induced by UV or cisplatin, which may be consistent with the differing repair kinetics of damage caused by the two treatments (Figs 3 and 5) and the different growth response of *T. brucei* cells following exposure to the two forms of damage and after RNAi against TbXPG (Figs 2 and 4).

### Predicted GG-NER proteins in *T. brucei* are involved in inter-strand cross-link repair

**Figure 8:**
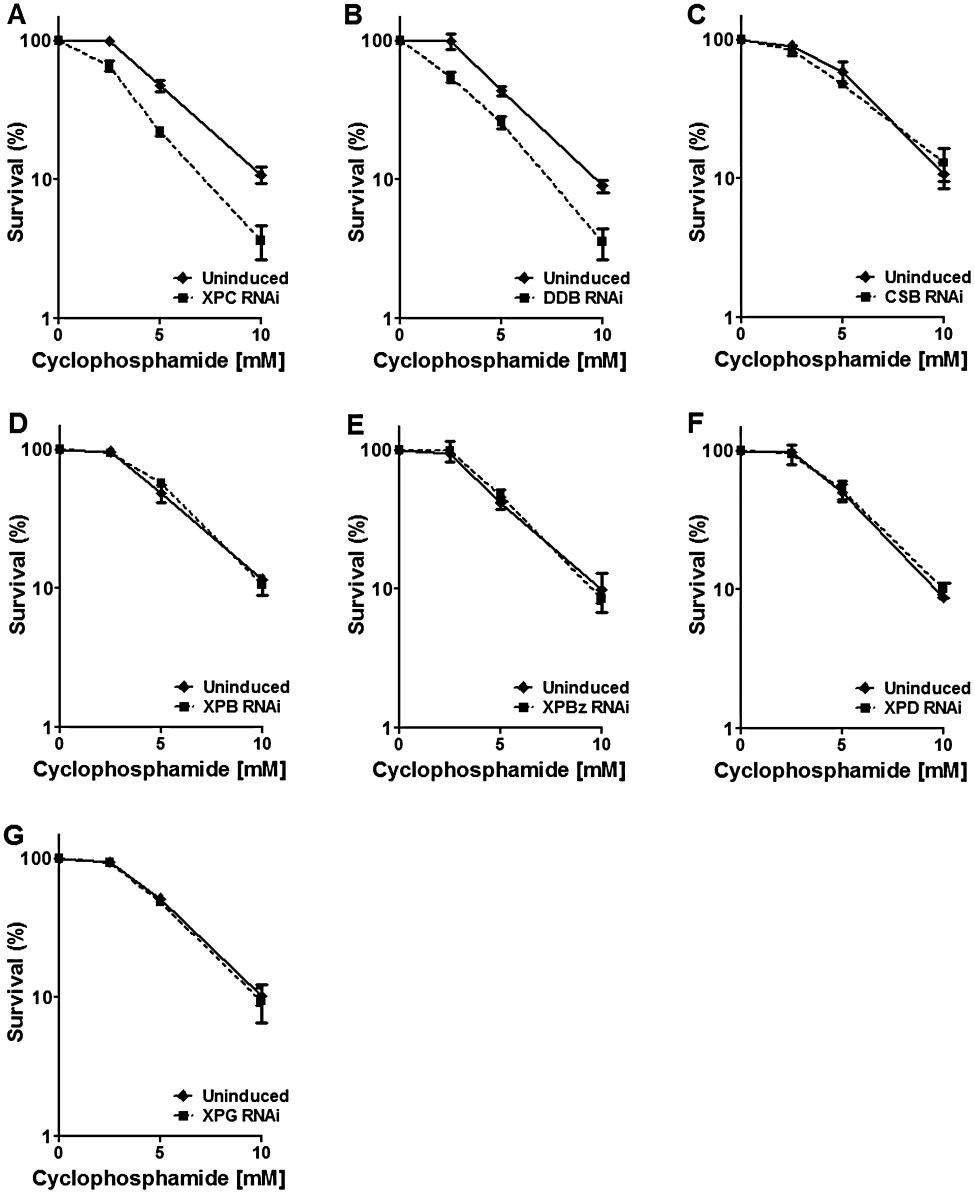
Predicted GG-NER proteins in *T. brucei* are involved in inter-strand cross-link repair. Survival curves of TbXPC, TbDDB, TbCSB, TbXPB, TbXPBz, TbXPD and TbXPD (A–G respectively) RNAi depleted cells (dotted lines) compared with respective uninduced controls (solid lines), after treatment with 2.5, 5 or 10 mM cyclophosphamide. Percent survival was calculated as the relative number of cells present in the treated groups compared with the untreated control. Values are means of three experiments, and vertical lines denote standard deviation.

Cyclophosphamide is an alkylating agent that adds monofunctional adducts at the N7 position of guanine and leads to the formation of DNA inter-strand cross-links (ICLs) involving two guanines (Povirk and Shuker, 1994). It has been used as a chemotherapeutic agent in a set of cancers and autoimmune diseases (Dollery, 1999). ICLs are processed in eukaryotes by the cooperation of three DNA repair and/or bypass pathways: NER, homologous recombination and translesion synthesis (McVey, 2010). To investigate the role of the predicted *T. brucei* NER genes during ICL repair, the BSF RNAi cell lines were cultivated in the presence of different doses of cyclophosphamide and survival measured before and after RNAi induction (Fig. 8). RNAi against TbXPC and TbDDB resulted in increased sensitivity to cyclophosphamide, indicating that these genes could be important in ICL repair. In contrast, RNAi depletion of TbCSB, TbXPBz, TbXPG, TbXPD or TbXPB had no effect on cyclophosphamide sensitivity. These data suggest that the separation of function we observed between the predicted GG-NER and TC-NER proteins in *T. brucei’s* response to UV or cisplatin is mirrored in the response to the cyclophosphamide: though TbXPC and TbDDB act in ICL repair, TbCSB, TbXPBz and TbXPG do not. Furthermore, these data add to the UV and cisplatin survival curves to provide more evidence that TbXPB or TbXPD do not act in DNA repair.

## Discussion

Much of the genome of trypanosomatids appears to be transcribed continuously, since most genes are arranged in multigene units, each transcribed from a single promoter (Vanhamme and Pays, 1995; Siegel *et al*., 2009;2011). This unusual mode of gene expression prompted us to investigate how NER operates, and in this study we have presented a near comprehensive functional analysis of the predicted machinery of NER in *T. brucei*. Our findings suggest that NER in *T. brucei*, and therefore most likely in related trypanosomatids, has undergone extensive functional diversification relative to other eukaryotes. Despite conservation of most predicted NER proteins, our data indicate that only TbCSB, TbXPBz and TbXPG contribute to UV and/or cisplatin repair, suggesting that TC-NER predominates. TbXPC and TbDDB, which would be expected to mediate GG-NER, show no evidence for a role in UV or cisplatin repair. Despite this, RNAi indicates that these factors are essential and act in ICL repair, suggesting that the GG-NER subpathway acts predominantly in an unknown but critical process in trypanosomatid genome maintenance. Finally, TbXPB and TbXPD show no evidence for a role in repair of any lesions tested, suggesting that *T. brucei* NER does not involve the TFIIH complex. Taken as a whole, this work suggests that genome maintenance by NER in this parasite may be quite unlike that of most eukaryotes: contrary to the expectation that the TC-NER and GG-NER subpathways should act in parallel and feed into a common lesion excision pathway, it appears that *T. brucei* has evolved a profound focus on TC-NER and has largely or completely uncoupled the GG-NER factors and TFIIH from this reaction.

### Does NER in *T. brucei* utilize a simplified machinery compared with other eukaryotes?

The analytical work reported here and elsewhere (see below) suggests that predictions of NER conservation in *T. brucei* based on homology masks a streamlining of the machinery devoted to NER, as well as neofunctionalization. A major element of NER streamlining is non-engagement of the TFIIH complex during the reaction, which appears to be associated with the evolution of a repair-specific XPB-related helicase, TbXPBz (or TbXPBr; Badjatia *et al*., 2013). Here we show that loss of TbXPBz by RNAi does not affect *T. brucei* viability, but that such RNAi renders BSF *T. brucei* cells sensitive to UV and cisplatin damage, showing that TbXPBz acts in DNA repair. In contrast, RNAi of TbXPB or TbXPD was lethal, but we found no evidence that loss of either helicase increased sensitivity to DNA damage. These findings are consistent with other studies. Lecordier *et al*. (2007) reported very similar rates of cell death following RNAi of TbXPB or TbXPD in procyclic form (PCF) and BSF cells, and Badjatia *et al*. (2013) have demonstrated that null mutants of *TbXPBz* can be generated in PCF *T. brucei* and display the same increased sensitivity to UV and cisplatin as TbXPBz RNAi-induced BSF cells (this study). Thus, the distinct functions of the related helicases TbXPBz and TbXPB appear to be retained throughout the parasite life cycle. Moreover, though TbXPB and TbXPD act in concert as components of the *T. brucei* TFIIH complex and are important in transcription (Lecordier *et al*., 2007), there is no evidence that TbXPBz associates with the TFIIH complex or contributes to transcription (Lee *et al*., 2009; Badjatia *et al*., 2013).

TbXPBz orthologues in trypanosomatids retain all conserved domains related to DNA helicase function (Lecordier *et al*., 2007; Badjatia *et al*., 2013), suggesting that XPB duplication arose to provide helicase-related activities for DNA repair that replace, or assume greater importance than, TbXPB-TFIIH. Though it has been proposed from sequence alignments that TbXPBz is capable of interacting with TbXPC and thus mediating GG-NER (Badjatia *et al*., 2013), the lack of evidence for TbXPC (or TbDDB) involvement in NER appears inconsistent with this (see below). In contrast, the phenotypes of TbXPBz RNAi strongly phenocopy those of TbCSB RNAi, suggesting that TbXPBz predominantly acts together with TbCSB in TC-NER. Whether or not this aspect of NER specialization is specific to kinetoplastids, or is also found in the wider range of protists that possess two XPBs (Badjatia *et al*., 2013), is unknown. Moreover, what aspects of TbXPBz prevent its recruitment into TFIIH and favours its association with TbCSB are unknown. Indeed, how TC-NER in *T. brucei* might operate without engaging TFIIH is unclear. In other eukaryotes, XPB is a weak helicase and ablation of this activity does not impede NER, while loss of XPD helicase is detrimental to NER (Coin *et al*., 2007), suggesting that XPD plays the greater role in extrusion of the lesion strand. Given these observations, the question of whether TbXPBz associates with another helicase in a functionally distinct variant of the TFIIH complex, lacking TbXPB and TbXPD, or acts in an unrelated complex, merits further investigation. In this light, the observations that TbXPBz interacts with Tbp52 (Badjatia *et al*., 2013), and that RNAi of Tbp52 or Tbp44 results in MMS and/or UV light sensitivity (Lecordier *et al*., 2007; Badjatia *et al*., 2013), are intriguing. One interpretation of these data is that at least some components of trypanosomatid TFIIH act in the DNA damage response, perhaps consistent with remodelling to incorporate TbXPBz and exclude TbXPB and TbXPD. However, sequence analysis of TbXPG provides further evidence that *T. brucei* NER has evolved to act without TFIIH.

We have shown that RNAi of TbXPG leads to increased sensitivity to cisplatin, indicating a role in TC-NER that would be consistent with work in other eukaryotes, where XPG has been shown to interact with CSB and play an early role in this NER subpathway (Iyer *et al*., 1996; Sarker *et al*., 2005). However, the polypeptide sequence of TbXPG displays notable sequence losses in the central spacer region relative to XPG orthologues (Fig. S7). Since the spacer region mediates the interaction of XPG with TFIIH (Dunand-Sauthier *et al*., 2005), mutations in this part of parasite protein suggest that engagement of TbXPG during NER is achieved without TFIIH. Indeed, lack of *T. brucei* TFIIH function in NER may underlie the potential absence of an identifiable trypanosmatid XPA orthologue, whose role appears to be in stabilizing TFIIH association with the NER machinery (Krasikova *et al*., 2010). Superficially, this putative bypass of TFIIH appears reminiscent of archaea, where XPB can interact with Bax1, a functional equivalent of the XPG nuclease (Rouillon and White, 2011). However, direct interaction between TbXPBz and TbXPG has not been observed, and a static complex appears incompatible with the differing response to UV exposure following RNAi against the two factors in *T. brucei* (discussed below). In addition, whether or not further endonucleases, such as XPF/ERCC1, contribute to NER in *T. brucei* awaits further investigation.

### Is translesion RNA synthesis an important alterative to TC-NER in *T. brucei*?

A number of observations lead us to propose that *T. brucei* may not always respond to transcription-blocking lesions by executing TC-NER. First, RNAi against TbCSB and TbXPBz resulted in increased sensitivity to both UV and cisplatin, whereas RNAi against TbXPG only caused increased sensitivity to cisplatin, not UV. Second, measuring repair kinetics shows that UV lesions are repaired substantially more slowly than cisplatin lesions in trypanosomes, and that TbCSB is needed to repair the latter type of damage. Finally, analysis of a number of genes spanning a *T. brucei* multigene transcription unit indicates differing changes in mRNA abundance following exposure to UV or cisplatin and after RNAi against TbCSB: increased mRNA levels were seen for promoter-distal genes after UV exposure, but reduced levels for the same genes after cisplatin. We suggest that these observations are most simply explained by RNA Pol II bypass of some lesions in some circumstances, and that CSB plays a pivotal role in the decision to invoke such a bypass or to enact TC-NER (Lee *et al*., 2002). Translesion synthesis by any RNA Pol has not been documented in trypanosomatids, but is well established in other eukaryotes. Lesions occurring in transcribed DNA strands lead to RNA Pol II blockage or slowdown, increasing CSB affinity with TFIIH, a recruitment that mediates the preferential repair of lesions localized in transcribed strands through TC-NER (Hanawalt and Spivak, 2008). Alternatively, DNA lesions present in the template strand can be bypassed by RNA Pol II (Saxowsky and Doetsch, 2006; Bregeon and Doetsch, 2011). CPDs can be bypassed during transcription because RNA Pol II has the ability to incorporate A residues in front a CPD dimer: the first A is incorporated in a non-template fashion, following the ‘A-rule’, while the second is inserted in a template-directed manner. When one U is incorporated instead of the second A, this mismatch induces RNA Pol II stalling. Thus, at least in *S. cerevisae*, the complete translesion transcription process through CPDs can be error-free (Brueckner *et al*., 2007; Walmacq *et al*., 2012). Though more work is needed, we suggest that the choice between translesion RNA synthesis and TC-NER is able to explain all the data we present.

We suggest that UV damage in *T. brucei* can be bypassed by translesion synthesis or removed by NER, and that TbCSB is pivotal in detecting such lesions and channelling them to TC-NER only if needed. Bypass of such lesions may normally be favoured due to multigenic transcription, avoiding accumulation of stalled RNA Pol II and promoting continued movement of the transcription machinery. In fact, this may be the reason that TFIIH is uncoupled from *T. brucei* NER, thus limiting CSB’s ability to engage NER. A preference for UV lesion bypass would explain why we see little evidence for rapid repair of UV damage. When TbCSB is removed, this control point is lost and TC-NER cannot occur, meaning that bypass predominates further and explaining why mRNA levels increased after TbCSB RNAi. It is likely that lesion bypass is a short-term response to minimal damage, and might explain the increased survival we observed in *T. brucei* BSF TbCSB RNAi-induced cells within the first four hours after UV exposure (Fig. S10). However, if bypass transcription is error-prone, it could lead to an overproduction of mutated mRNAs that could be toxic for the parasite, explaining the increased sensitivity in TbCSB RNAi cells 24 h after UV irradiation. We further suggest that cisplatin-induced adducts have a more pronounced effect on *T. brucei* transcription than UV, inducing RNA Pol II blockages that cannot be as efficiently bypassed (Damsma *et al*., 2007). Thus, the primary response to cisplatin, and perhaps other platinum compounds, is that TbCSB signals for lesion removal by enacting TC-NER and engaging TbXPG. This model explains why TbXPG RNAi cells are more sensitive to cisplain, but not UV, and why cisplatin is seen to be rapidly removed in the repair assay. The model also explains why cisplatin treatment results in decreased mRNA levels of most genes in the polycistron after TbCSB RNAi: here, TC-NER is impaired and cisplatin adducts cause increasing transcription impairment through polycistons.

### Why is loss of some predicted *T. brucei* NER factors lethal?

We have argued above that NER in *T. brucei* is largely conducted by a simplified TC-NER pathway, involving TbCSB, TBXPBz and TbXPG. In this light, the data that we present on the functions provided by the predicted *T. brucei* GG-NER factors are remarkable, since RNAi of TbXPC or TbDDB results in cell death, at least in the BSF. The essentiality of TbXPB and TbXPD has been documented before in *T. brucei* (Lecordier *et al*., 2007) and is explained by these factors’ roles as components of the TFIIH complex; hence, their removal impedes transcription, as seen by reduced mRNA levels following RNAi (this study) and evaluated by *in vitro* transcription assays (Badjatia *et al*., 2013). In contrast, why the GG-NER factors are essential is less clear. In *S. cerevisiae*, null mutants of *RAD4* (XPC), *RAD23*, *RAD1* (XPF) and *RAD10* (ERCC1) have been documented and are viable (Giaever *et al*., 2002). Phenotypes of NER mutation appear more severe in multicellular eukaryotes but, even here, at least some NER components appear to be dispensable. In *A. thaliana* XPF/ERCC1 null mutants are viable (Vannier *et al*., 2009), while in mice substantial gene deletions of XPC are viable (Sands *et al*., 1995) and a near complete null of ERCC1 does not prevent embryo growth until after gestation. In *C. elegans*, loss of RAD23 or XPC is not immediately lethal, with the former ablation allowing complete embryonic development but maternal sterility (Kamath *et al*., 2003). The essentiality of TbDDB and TbXPC in *T. brucei* appears not to be due to critical contributions to NER, since we find no evidence that TbDDB or TbXPC repair UV or cisplatin damage. However, this DNA damage may have the most pronounced effect in the highly transcribed core of the *T. brucei* genome, and we cannot yet discount that GG-NER is important in the subtelomeres, which harbour thousands of VSG genes and are largely transcriptionally silent (Berriman *et al*., 2005; Marcello and Barry, 2007). Nonetheless, we have provided preliminary evidence that GG-NER may have assumed a predominant role in non-NER DNA repair.

**Figure 9:**
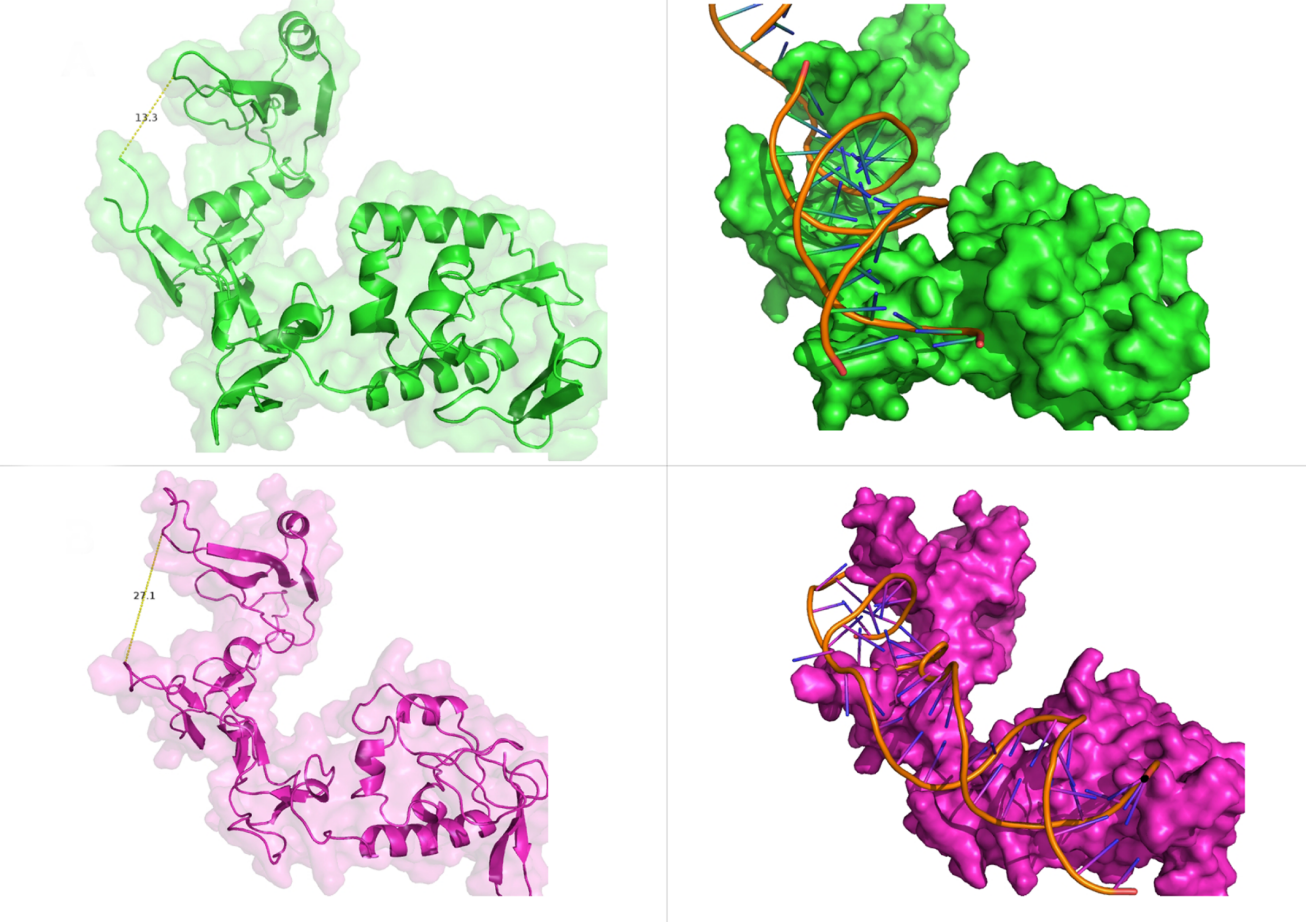
TbXPC presents a larger DNA damage recognition site when compared with its yeast homologue. Left: a comparison of the structure of Rad4 (green) and the predicted structure of TbXPC (purple); secondary structures are shown, overlapped with the surface, and the distance difference between the ‘arms’, which constitutes the DNA lesion recognition site, of both proteins is depicted. Right: Surface representation of the structure of Rad4 (green) and the predicted structure of TbXPC (purple), bound to a double-stranded DNA molecule.

The lack of recognition of UV-induced lesions by TbXPC and TbDDB is remarkable, since UV-induced lesions are recognized by XPC and XPE during GG-NER in most studied organisms (Feldberg and Grossman, 1976; Chu and Chang, 1988). Rad4, the *S. cerevisiae* homologue of XPC, recognizes (together with RAD23) ssDNA structures associated with bulky lesions. To do this, a β-hairpin domain of Rad4 is inserted through the DNA double helix, evicting the damaged strand away from its binding site. This mechanism of XPC DNA damage recognition accounts for the remarkable range of bulky lesions targeted by GG-NER (Min and Pavletich, 2007). To ask if the lack of UV damage repair by GG-NER may be accounted for by alterations in TbXPC, the three dimensional structure of the parasite protein was modelled on the structure of Rad4 protein bound to DNA (Fig. 9). By measuring the distance between the ‘arms’ that form the lesion recognition site in the two proteins, we calculated the approximate size of the DNA binding site to be around 13 Å for Rad4 and around 27 Å for TbXPC. Since the diameter of a DNA double helix is approximately 20 Å, it is plausible to infer that Rad4 can only recognize one DNA strand, while TbXPC may bind both strands. This modelling raises the possibility that TbXPC can bind both damaged and undamaged DNA strands, and might explain why GG-NER in trypanosomes is not targeted to DNA damage caused by UV and cisplatin. This putative alteration in TbXPC DNA binding may also be related to the more pronounced role we see for the *T. brucei* GG-NER proteins acting in ICL repair, based on the observation that RNAi of TbXPC or TbDDB caused sensitivity to the ICL inducer cyclophosphamide.

ICLs are highly genotoxic DNA lesions whose repair requires several factors derived from a number of independent DNA repair pathways (McVey, 2010). A role for GG-NER proteins in ICL repair has been seen in *S. cerevisiae* (Saffran *et al*., 2004), where *rad4*Δ mutants (XPC mutants) shows sensitivity to the ICL-causing agents mechlorethamine, mitomycin C and cisplatin (McHugh *et al*., 1999; Wu *et al*., 2004). However, mammal cells deficient for XPA or XPC present only moderate sensitivity to ICL-inductors (Clingen *et al*., 2007). High sensitivity to ICL-inducing agents, as well as bone marrow failure and high cancer frequencies, are observed in humans harbouring mutations in Fanconi Anemia core complex genes (Deans and West, 2011), which appear to be missing in lower eukaryotes. Thus, in single-celled eukaryotes like yeast and trypanosomatids ICL recognition and repair by GG-NER proteins may assume greater importance. However, if ICL repair is the main role of the GG-NER pathway in *T. brucei*, it remains unclear why this activity should assume such great importance. From the molecular modelling above, we infer a novel mechanism employed by TbXPC for DNA lesion binding relative to Rad4 (Min and Pavletich, 2007). However, it is unclear if this novelty is due to greater commitment of the *T. brucei* GG-NER system to ICL recognition, since Rad4 can act in ICL repair and, indeed, the human XPC-Rad23b complex also binds to a psoralen ICLs (Thoma *et al*., 2005). Further investigation will be necessary to address the lesion spectrum targeted by TbXPC and, until then, it remains unclear what essential purpose these GG-NER factors provide. Nonetheless, the possibility that TbXPC and TbDDB, previously thought to act in NER, might have assumed a distinct and essential genome maintenance role is interesting. If it can be shown that GG-NER provides an activity distinct from the host, this repair pathway might represent a target for therapeutic intervention against Sleeping Sickness and other trypanosomatid diseases.

## Experimental procedures

### Parasite growth

Epimastigote forms of *Trypanosoma cruzi* CL Brener strain were cultivated at 28°C in LIT (Liver Infusion Tryptose) medium (pH 7.3; Camargo, 1964) supplemented with 10% heat-inactivated fetal bovine serum (FBS, Cultilab) plus 100 U ml^−1^ penicillin and 100 μg ml^−1^ streptomycin (Invitrogen). Bloodstream form *Trypanosoma brucei* were cultivated at 37°C in a humidified incubator with 5% CO_2_. Parasites were grown in HMI-9 (Hirumi and Hirumi, 1989) supplemented with 20% heat-inactivated FBS (Sigma) plus 1% (v/v) of penicillin and streptomycin (Sigma).

### Growth after RNAi in the presence and absence of genotoxic agents

To measure growth, the parasites were diluted to a density of 1 × 10^5^ cells ml^−1^ in HMI-9 plus selective drugs (hygromycin and phleomycin), with and without tetracycline (1 μg ml^−1^, to induce RNAi). Cell cultures were diluted 10-fold daily and cell numbers counted in a heamocytometer chamber at 24 h intervals; each growth curve was carried out three times independently for each RNAi cell line. To evaluate sensitivity to DNA damage, the cells were similarly diluted to 1 × 10^5^ cells ml^−1^, and tetracycline added as before. After 24 h the cells were exposed to the genotoxic agents, and the cell growth allowed to proceed as before. Survival rate was calculated from the cell counts of the drug-exposed cells relative to the untreated controls. All survival curves were performed in triplicate. For the cisplatin survival curve, parasites were treated with 1, 2 or 5 μM of cisplatin (Sigma). In the UV irradiation survival curve, parasites received doses of 500, 1500 or 3000 J m^−2^ of UVC using a *Stratalinker® UV Crosslinker* (Stratagene). For cyclophosphamide (Sigma) exposure, parasites were grown in concentrations of 2.5, 5 and 10 mM.

### RNAi

The primers utilized for RNAi constructs were designed using software available online (http://trypanofan.path.cam.ac.uk/software/RNAit.html); in each case Attb1 and Attb2 recombination sites were added to the termini of the primers. The sequences of the primers used to PCR-amplify the RNAi cassettes are on Table S1. All primers were purchased from Eurofins MWG Operon (http://www.eurofinsdna.com). The Attb1 and Attb2 sites allowed Gateway recombination in a single reaction, inserting two copies of the product in opposing directions into the pGL2084 vector to generate a stem-loop RNAi producing construct (Jones *et al*., 2014). For PCR, a master mix was used: 1× Phusion Buffer, 0.2 mM dNTP mix, 50 ng of template genomic DNA from Lister 427 *T. brucei*, 2 μM of each primer and 20 u ml^−1^ of Phusion enzyme (Invitrogen). The PCR reactions were carried out with settings of 3 min at 98°C, 30 cycles of 98°C for 30 s, 60°C for 30 s and 72°C for 30 s, and a final step for 10 min at 72°C. PCR products were purified from 1% agarose gels using the Qiagen MinElute Kit and used in RNAi construct assembly into pGL2084. For the assembly reaction ≥ 10 ng of PCR product, 150 ng of pGL2084 vector, 0.25 μl of BP Clonase™ enzyme mix (Invitrogen), Tris-HCl 3.75 mM and EDTA 375 μM (pH 8.0) were used. The mix reaction was incubated at room temperature for 1 h. To transform Max efficiency DH5α *E. coli* (Invitrogen Cat. No. 18258-012), 1 μl of BP clonase reaction was used, as described by the manufacturer. The clones obtained for each reaction were grown in LB medium supplemented with ampicilin at 100 μg ml^−1^ at 37°C overnight and the RNAi constructs were then isolated using Qiagen QIAprep Spin Kit. To test if the extracted plasmids were successfully recombined, double digestions with BamHI and XbaI, and single digestions with StuI or ClaI, were performed. Next, the RNAi constructs were linearized by digestion with AscI and 5 μg of linearized plasmid were used to transfect BSF *T. brucei* 2T1 cells (Alsford and Horn, 2008). For transfection 1 × 10^7^ cells were harvested by centrifugation for 10 min for 1500 *g*. After media was removed, cells were resuspended in 100 μl of Amaxa Nucleofector T-cell buffer, transferred to a cuvette and 10 μl of the linearized DNA were added. Electroporation was performed using the program X-001 on the Nucleofector II machine. Cells were then transferred to 30 ml of HMI-9 medium without selective drugs. After 6 h, the cells were diluted at 1:20 and 1:40 in media containing selective drugs: hygromycin (2.5 μg ml^−1^) and phleomycin (0.5 μg ml^−1^). Antibiotic resistant clones were selected for at least 7 days. In order to test if the RNAi cassette had been correctly integrated, the parasites’ sensitivity to puromycin (1.0 μg ml^−1^) was tested.

### Real-time PCR

To validate the RNAi, levels of target mRNAs for each gene were measured by quantitative reverse transcriptase PCR (qRT-PCR). A total of 2 × 10^7^ cells were harvested from RNAi-induced and uninduced cultures at the times stated, and total RNA was isolated using the Qiagen RNeasy Kit (with on-column DNaseI digestion). For all qRT-PCRs a master mix for 30 reactions was made in which each reaction had 12.5 μl of SYBR Green PCR Master Mix (Applied Biosystems), 1.0 μl of each primer (300 nM stock), 9.5 μl of dH_2_O, and 1.0 μl cDNA (generated by SuperScript First-Strand Synthesis System for RT-PCR (Invitrogen) from the total RNA, according to manufacturer’s instructions). Reactions were run on an ABI Prism 7000 thermocycler and mRNA levels quantified from the amplifications according to the manufacturer’s instructions; conditions for all reactions were 50°C for 2 min, 95°C for 10 min, followed by 40 cycles of 95°C for 15 s and 60°C for 1 min. Primers recognizing *GPI8* (CTOL27 and CTOL28) were used as a control (Tiengwe *et al*., 2012). Primer pairs for each putative NER gene are provided on Table S1. The mRNA levels from the polycistron genes were also assessed by qRT-PCR (for primers details, see Table S1), using the same procedures. All primers were purchased from Eurofins MWG Operon. All qRT-PCR experiments were performed at least three times independently. Statistical analysis was performed using unpaired *t*-test for comparisons of mRNA levels between RNAi cell lines and uninduced controls. An ordinary one-way anova followed by Tukey’s test was performed to compare mRNA levels after 24–48 h of RNAi induction, while a two-way anova followed by Tukey’s test was performed to compare mRNA levels after RNAi induction in the presence of DNA damage. All statistical analysis was conducted using the software GraphPad Prism.

### Comparative modelling and docking

A TbXPC molecular structure was generated by comparative modelling. The search for candidate template structures was performed by BLAST searches of the PDB database (Westbrook *et al*., 2003). The template used for modelling was Rad4 2QSH chain A (*S. cerevisiae* XPC homologue; Min and Pavletich, 2007). The TbXPC molecular model was built based only on the β-hairpin domains of Rad4, corresponding to positions 400–700 of the full-length protein. Sequence alignments between TbXPC and its structural template were generated by Promals 3D (Pei *et al*., 2008) and manually curated. Molecular modelling was performed using the program Modeller (version 9.7; Eswar *et al*., 2007), which generated a hundred possible structures. The validation of obtained models according to stereochemical quality was performed through the analysis of Ramachadran plots generated by the program Procheck (version 3.5.4; Morris *et al*., 1992) and the energetic characteristics were assessed using ProSA (ProSa 2003; Wiederstein and Sippl, 2007). Perl scripts were designed and used to automatically retrieve the percentage of residues in the most favoured regions of the Ramachandran plot and the Z-score of ProSA for each one of the 100 structures. Three different types of software were used to determine potential protein-DNA binding sites: DNABindR, BindN (and its variant BindNPlus) and DBSPSSM (Ahmad and Sarai, 2005; Wang and Brown, 2006; Yan *et al*., 2006). Results were clustered and all amino acids predicted as DNA-binding by at least one of the three algorithms were considered as active residues during docking experiments. Docking calculations were carried out in Haddock (Dominguez *et al*., 2003) advanced guru interface, defining active residues in the proteins as described above, and defining passive residues as all residues within a radius of 6 angstroms around the active residues. For the DNA molecules, all nucleotides were considered as active residues in protein-DNA interaction. All additional parameters were used as default values. The generated complexes were analysed through stereochemical, cluster-sizes and energy evaluations.

### Immunofluorescence

Approximately 1 × 10^6^ cells were collected, washed once in PBS, and allowed to attach for 4 min to 12-well multi-well glass slides (Thermo Scientific) that had been previously coated with Poly-L-lysine (Sigma Aldrich). The cells were then fixed with 4% formaldehyde in PBS for 15 min, washed twice with PBS, incubated with 0.5% Triton X-100 for 20 min, and further washed with PBS (twice). Next, the cells were incubated for 30 min with 2 M HCL, washed 5 times with PBS, and then blocked with 20% heat-inactivated FBS (Sigma-Aldrich) for 30 min at 37°C, after which the wells were washed 5 times with PBS. Primary antisera were used according to the manufacturer instructions: mouse anti-(6-4) phosphoproducts (6-4 PPs) antibody (Cosmo Bio Co., Ltd) at a 1:300 dilution, mouse anti-cyclobutane pyrimidine dimers (CPDs) antibody (Cosmo Bio Co., Ltd) at a 1:1500 dilution, and mouse anti-cisplatin antibody (Abcam) at a 1:400 dilution. In all cases, the antiserum was added to the cells in PBS containing 5% FBS, and incubated for 30 min at 37°C. The cells were then washed 5 times with PBS, and further incubated for 30 min at 37°C with Alexa Fluor 594 goat anti-mouse IgG (H+L) (Invitrogen) antibody at a dilution of 1:100 in PBS containing 5% FBS. The wells were then washed 5 times with PBS and the slides mounted in VECTASHIELD mounting medium with DAPI (1.5 μg ml^−1^) (Vector Laboratories). The slides were examined, and images acquired, using an Axioskop 2 fluorescence microscope (Zeiss). Images were processed and assembled in Adobe Photoshop (Adobe Systems). A two-way anova followed by a Bonferroni post-test was performed to compare the levels of cisplatin-induced damage in experimental repeats comparing TbCSB RNAi induced cells and uninduced controls. Statistical analysis was conducted using the software GraphPad Prism.

### DNA lesion detection through quantitative PCR

To detect the level of DNA damage in the nuclear genome, a quantitative PCR-based technique was used according to previously described protocols (Santos *et al*., 2006). Briefly, lesion frequency was assessed through relative PCR-amplification of a 10 kb fragment of the *T. brucei* or *T. cruzi* genome, comparing amplification after treatment with genotoxic agents relative to untreated controls. A small, internal DNA fragment, comprising 204 and 250 bp (in *T. brucei* an *T. cruzi* respectively) was PCR-amplified from the 3′ extremity of the larger molecule in order to correct for DNA loading errors or PCR bias and assure that amplification conditions were the same for all samples. For DNA damage induction in *T. brucei*, approximately 1 × 10^8^ BSF cells of strain Lister 427 were harvested by centrifugation for 15 min at 3000 *g* at 4°C and resuspended in the same volume of 1× PBS. Next, cells were exposed to 100 μM cisplatin for 1 h. For UVC exposure, 1 × 10^8^ parasites were concentrated in 5 ml of media. The cells were then spread in a small Petri plate and exposed to 1500 J m^−2^ of UVC in a *Stratalinker® UV Crosslinker* (Stratagene). For DNA damage induction in *T. cruzi*, 1 × 10^8^ epimastigote form cells, CL Brener strain, were used. Parasites were centrifuged as for *T. brucei* and treated with 300 μM of cisplatin in 1× PBS for 1 h. For UV treatment, 5 ml of culture containing 1 × 10^8^ *T. cruzi* cells were spread in a small Petri plate and irradiated with 1500 J m^−2^ of UVC. In all cases, the cells were centrifuged after treatment (as before) and resuspended in conditioned medium, i.e. the medium used in the original culture. Untreated samples were processed in the same way. Samples were collected at appropriate time points subsequent to the above treatments (0, 1, 2, 5 and 10 h after treatment for *T. brucei*, and 0, 1, 5 and 24 h for *T. cruzi*), centrifuged at 3000 g and 4°C for 15 min, and the resulting cell pellet immediately frozen at −80°C. DNA was prepared from the cells using the Blood & Cell Culture DNA Mini Kit (QIAGEN), according to the manufacturer’s instructions for DNA extraction from tissues. DNA was then quantified using PicoGreen dye (Molecular Probes) and standard curves, as described by Santos *et al*. (2006). PCR was performed from 15 ng of template DNA using the GeneAmp XL PCR Kit (Applied Biosystems) and the resulting amounts of PCR product quantified, again using PicoGreen and as described by Santos *et al*. (2006). The primers used in the amplification reaction of the large fragment in *T. brucei* were qPCRFoward (5′-GTTGCTCACTTTCACCACGTATTCGGGAACCTGT-3′) and qPCRReverse (5′-CCACTGAATGCTGTATCCGGCATTTAGTCGTGTCTATGGG-3′); for *T. cruzi* the large fragment used the primers QPCRNuc2F (5′-GCACACGGCTGCGAGTGACCATTCAACTTT-3′) and QPCRNuc2R (5′-CCTCGCACATTTCTACCTTGTCCTTCAATGCCTGC-3′). To PCR-amplify the small fragment, used as the internal control, the primers qPCRFI (5′-TTACAGCACCCAGGTTTATACCGCACGAAAGTGG-3′) and qPCRReverse were used in *T. brucei*, while the primers QPCRNuc2Int (5′-TCGAGCAAGCTGACACTCGATGCAACCAAAG-3′) and QPCRNuc2R were used in *T. cruzi*. PCR reactions must to be carried out only until the logarithmic phase, when the amplification yields are directly proportional to the starting amount of template. To meet these requirements, PCR conditions in this experiment need to optimized for each lab, as they are dependent on many factors, including the type of PCR machine used and the accuracy of genomic DNA template quantification using the PicoGreen dye system (see Santos *et al*., 2006 for guidelines and for the quantification strategy).
