# Supplementary material for: Nucleotide excision repair in Trypanosoma brucei: specialization of transcription-coupled repair due to multigenic transcription
Source: Mol Microbiol. 2014 Apr 24;92(4):756–76. doi: 10.1111/mmi.12589 (PMC4138998; doi:10.1111/mmi.12589)
Supplement: Supplementary file 1 [file mmi-92-756-sd1.pdf]

Nucleotide excision repair in *Trypanosoma brucei*: specialization of transcription-coupled repair due to multigenic transcription.

Carlos R. Machado [1#*], João P. Vieira-da-Rocha [1#], Isabela Mendes [1,3], Matheus A. Rajão [2], Lucio Marcello [3], Mainá Bitar [1], Marcela G. Drummond [5], Priscila Grynberg [1], Denise A. A. Oliveira [5], Catarina Marques [3], Ben Van Houten [4], Richard McCulloch [3*]

[1] Departamento de Bioquímica e Imunologia, ICB, Universidade Federal de Minas Gerais, Av. Antônio Carlos, 6627, Caixa Postal 486, Belo Horizonte 30161-970, MG, Brazil.

[2] Coordenação de Pesquisa, Instituto Nacional de Câncer, Rua André Cavalcanti, 37 Fátima 20231-050 - Rio de Janeiro, RJ, Brazil.

[3] The Wellcome Trust Centre for Molecular Parasitology, College of Medical, Veterinary and Life Sciences, Institute of Infection, Immunity and Inflammation, University of Glasgow, Sir Graeme Davies Building, 120 University Place, Glasgow, G12 8TA, UK.

[4] Department of Pharmacology and Chemical Biology, University of Pittsburgh School of Medicine and The University of Pittsburgh Cancer Institute, Hillman Cancer Center, Pittsburgh, PA 15213, USA.

[5] Laboratório de Genética Animal, Escola de Veterinária, Universidade Federal de Minas Gerais, Av. Antônio Carlos, 6627, Caixa Postal 486, Belo Horizonte 30161-970, MG, Brazil.

[#] These authors contributed equally to this work.

* Corresponding authors: richard.mcculloch@glasgow.ac.uk; FAX: +44(0)141 330 8269 and crmachad@icb.ufmg.br; FAX: (5531) 3409-2984

Supporting Figure legends

Supporting figure 1: *T. brucei* XPC. The upper diagram shows predicted conserved domains in TbXPC when the polypeptide is used to interrogate the proteins in the NCBI database by BLASTp analysis. The lower diagram shows a sequence alignment of TbXPC with related proteins from other eukaryotes; residues identical or conserved in >50% of sequences are shaded black or grey, respectively.

Supporting figure 2: *T. brucei* RAD23. The upper diagram shows predicted conserved domains in TbRAD23 when the polypeptide is used to interrogate the proteins in the NCBI database by BLASTp analysis. The lower diagram shows a sequence alignment of TbRAD23 with related proteins from other eukaryotes; residues identical or conserved in >50% of sequences are shaded black or grey, respectively.

Supporting figure 3: *T. brucei* DDB. A sequence alignment of TbDDB (only the larger DDB subunit could be identified) with related proteins from other eukaryotes; residues identical or conserved in >50% of sequences are shaded black or grey, respectively.

Supporting figure 4: *T. brucei* CSB. The upper diagram shows predicted conserved domains in TbCSB when the polypeptide is used to interrogate the proteins in the NCBI database by BLASTp analysis. The lower diagram shows a sequence alignment of TbCSB with related proteins from other eukaryotes; residues identical or conserved in >50% of sequences are shaded black or grey, respectively.

Supporting figure 5: *T. brucei* XPD. The upper diagram shows predicted conserved domains in TbXPD when the polypeptide is used to interrogate the proteins in the NCBI database by BLASTp analysis. The lower diagram shows a sequence alignment of TbXPD with related proteins from other eukaryotes; residues identical or conserved in >50% of sequences are shaded black or grey, respectively.

Supporting figure 6: *T. brucei* XPB homologues. The upper diagrams shows predicted conserved domains in TbXPB and TbXPBz when the polypeptides are used to interrogate the proteins in the NCBI database by BLASTp analysis. The lower diagram shows a neighbor-joining tree of the two *T. brucei* XPB homologues with related proteins from other eukaryotes and from an archaea.

Supporting figure 7: *T. brucei* XPG. The upper diagram shows predicted conserved domains in TbXPG when the polypeptide is used to interrogate the proteins in the NCBI database by BLASTp analysis. The middle diagram shows a summary of the comparative sizes and predicted conserved domains in XPG from *T. brucei* and from *H. sapiens*. The lower diagram shows a sequence alignment of TbXPG with related proteins from other eukaryotes; residues identical or conserved in >50% of sequences are shaded black or grey, respectively.

Supporting figure 8: *T. brucei* XPF. The upper diagram shows predicted conserved domains in TbXPF when the polypeptide is used to interrogate the proteins in the NCBI database by BLASTp analysis. The lower diagram shows a sequence alignment of TbXPF with related proteins from other eukaryotes; residues identical or conserved in >50% of sequences are shaded black or grey, respectively.

Supporting figure 9: *T. brucei* ERCC1. A sequence alignment is shown of TbERCC1 with related proteins from other eukaryotes; residues identical or conserved in >50% of sequences are shaded black or grey, respectively.

Supporting figure 10: TbCSB depletion increases UV-resistance for a short period post-irradiation. Survival curve of TbCSB depleted cell line and its uninduced control, 4 hours after treatment with 1500 J/m^2 of UVC. Percent survival was calculated as the relative percentage of cells present in the treated groups compared with the untreated control. Values are means of three experiments, and vertical lines denote standard deviation.

Table S1: Primers used in RNAi construct production and mRNA level quantification.

Primer	Sequence
XPBrnai_F	GGGGACAAGTTTGTACAAAAAAGCAGGCTAGCCACTGTTAAGAAGCGGA
XPBrnai_R	GGGGACCACTTTGTACAAGAAAGCTGGGTCTCCACTCGAGCAAGGAAAC
XPBzrnai_F	GGGGACAAGTTTGTACAAAAAAGCAGGCTACCGACGCTTGGTTTTACAC
XPBzrnai_R	GGGGACCACTTTGTACAAGAAAGCTGGGTGCCTACTAACCTTTGCAGCG
XPCrnai_F	GGGGACAAGTTTGTACAAAAAAGCAGGCTCCTTGTTGGGTAACCGCTAA
XPCrnai_R	GGGGACCACTTTGTACAAGAAAGCTGGGTCGCCCACAGAAAAGGTGTAT
XPDrnai_F	GGGGACAAGTTTGTACAAAAAAGCAGGCTTGACGGAGCTCAAGAAAGGT
XPDrnai_R	GGGGACCACTTTGTACAAGAAAGCTGGGTACCTTTAGCGCCTTCCTAGC
XPErnai_F	GGGGACAAGTTTGTACAAAAAAGCAGGCTATGCACCAGGGTAGTTTTGC
XPErnai_R	GGGGACCACTTTGTACAAGAAAGCTGGGTTTCTAGAGTCGGCCAAGCAT
XPFrnai_F	GGGGACAAGTTTGTACAAAAAAGCAGGCTGAATGGGAGCGATGGACTAA
XPFrnai_R	GGGGACCACTTTGTACAAGAAAGCTGGGTCCACTAAACGAACGGCATTT
ERCC1rnai_F	GGGGACAAGTTTGTACAAAAAAGCAGGCTTGTGTTGTGCTTCTTCTCCG
ERCC1rnai_R	GGGGACCACTTTGTACAAGAAAGCTGGGTTCATAAATGCAGCGAAGTGC
XPGrnai_F	GGGGACAAGTTTGTACAAAAAAGCAGGCTACCTGGTGAAAGTGTGGAGG
XPGrnai_R	GGGGACCACTTTGTACAAGAAAGCTGGGTAACCTCCCGAGTTCGTTTTT
CSBrnai_F	GGGGACAAGTTTGTACAAAAAAGCAGGCTACCGGAGAAATACGAACGTG
CSBrnai_R	GGGGACCACTTTGTACAAGAAAGCTGGGTCTGTTACTGCGCATCGAAAA
XPCrnaq_F	GAAGTCGCTGCAGCCAAGA
XPCrnaq_R	GCGTCAGCGAAGTCATCAAG
XPEFrnaq_F	GAATTGATTTCCGTGACGTTTCT
XPERrnaq_R	CGGCGACCGCATTTTC
XPBzrnaq_F	TAACGTTAACATTGCGTTGAAGTCT
XPBzrnaq_R	GCGTCAACAGCAGCGATCT
XPBrnaq_F	AGTTGGTGGAGGGCAATCC
XPBrnaq_R	AGCAAGCGGTCCACAACAG
XPDrnaq_F	GACTGACCAGCGTTGTGTGTACTT

XPDrnaq_R	CGGGCGGAGATCAAAGC
XPGrnaq_F	CCAGCGCCGTCAACCA
XPGrnaq_R	AAGTCGTCGTTGAGCAGAATCA
XPFrnaq_F	TCCCGACTGGACCCCTTATC
XPFrnaq_R	GGGTTGACCTCCGGAACAG
ERCC1rnaq_F	CGGAACTGCCGGGTTTC
ERCC1rnaq_R	CGTTAAGTACGGCGTGAAGTCTT
CSBrnaq_F	GAGCACTGCCGGTTGCTT
CSBrnaq_R	TGCCCCTCGTCAAGAATAACA
HIPB1rnaq_F	ACGCTGCAAATTATGCTTTACG
HIPB1rnaq_R	GGCACGTCTATCTCCAAAGAAAA
MANB2rnaq_F	CGGCTCGCTGTGGATCTT
MANB2rnaq_R	CCCAGTGGGCGTGTCAA
TCPB3rnaq_F	GACCTCGCGGCTCACTTC
TCPB3rnaq_R	CTTACGCAAACGGCGAATG
HIP2B4rnaq_F	GCACAGGCGGGAAACG
HIP2B4rnaq_R	ATCCCCATGTCGGCAGAA
ACTA1rnaq_F	CGGCTCGTGGGCTTTAAAG
ACTA1rnaq_R	ACGCCGCCGTAAGTTCTG
DNATA3rnaq_F	CGTGAGGAGTTGCCACAGAA
DNATA3rnaq_R	TACGGCACCCACTCCTGAA
HIP3A4rnaq_F	GTTGAGCGAATGTGCATGCT
HIP3A4rnaq_R	CGCGCCGAAGTGTAAAGC
HIP4A5rnaq_F	CGTTGAAGACCGCGCAGTA
HIP4A5rnaq_R	GAGGCGCAGTTCAAGCATCT
GPI8rnaq_F	TCTGAACCCGCGCACTTC
GPI8rnaq_R	CCACTCACGGACTGCGTTT

All primer names contain the name of target gene (XPE refers to DDB). “rnai” label is designed for primers used in production of RNAi constructs, while “rnaq” label is for all primers utilized in mRNA quantification analysis. “_F” and “_R” mean primer forward and reverse, respectively.

T. brucei XPC

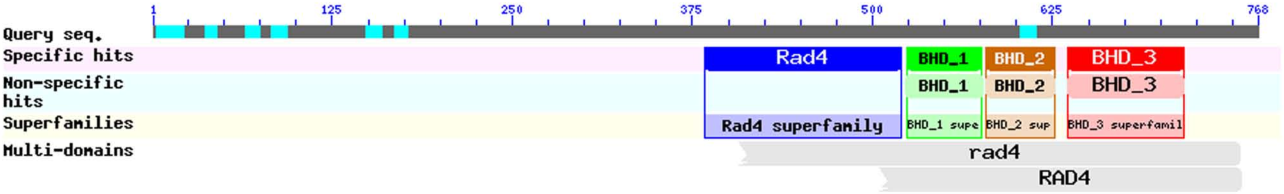

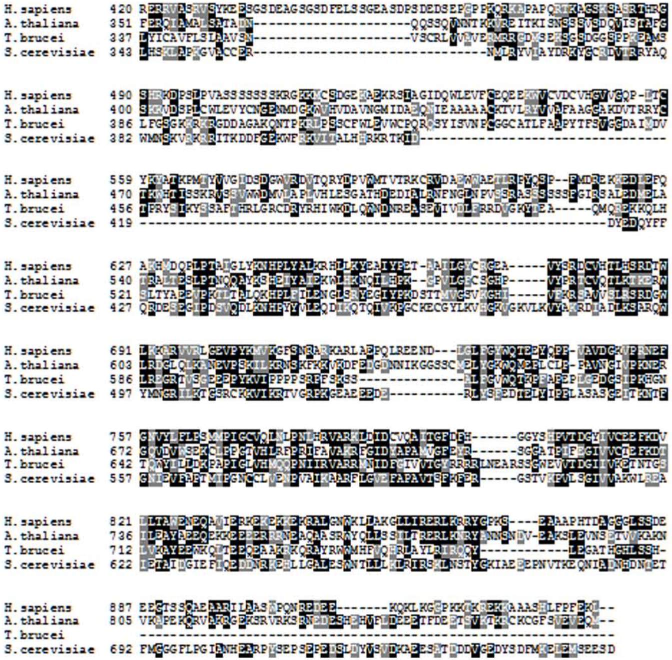

Fig.S1

T. brucei RAD23

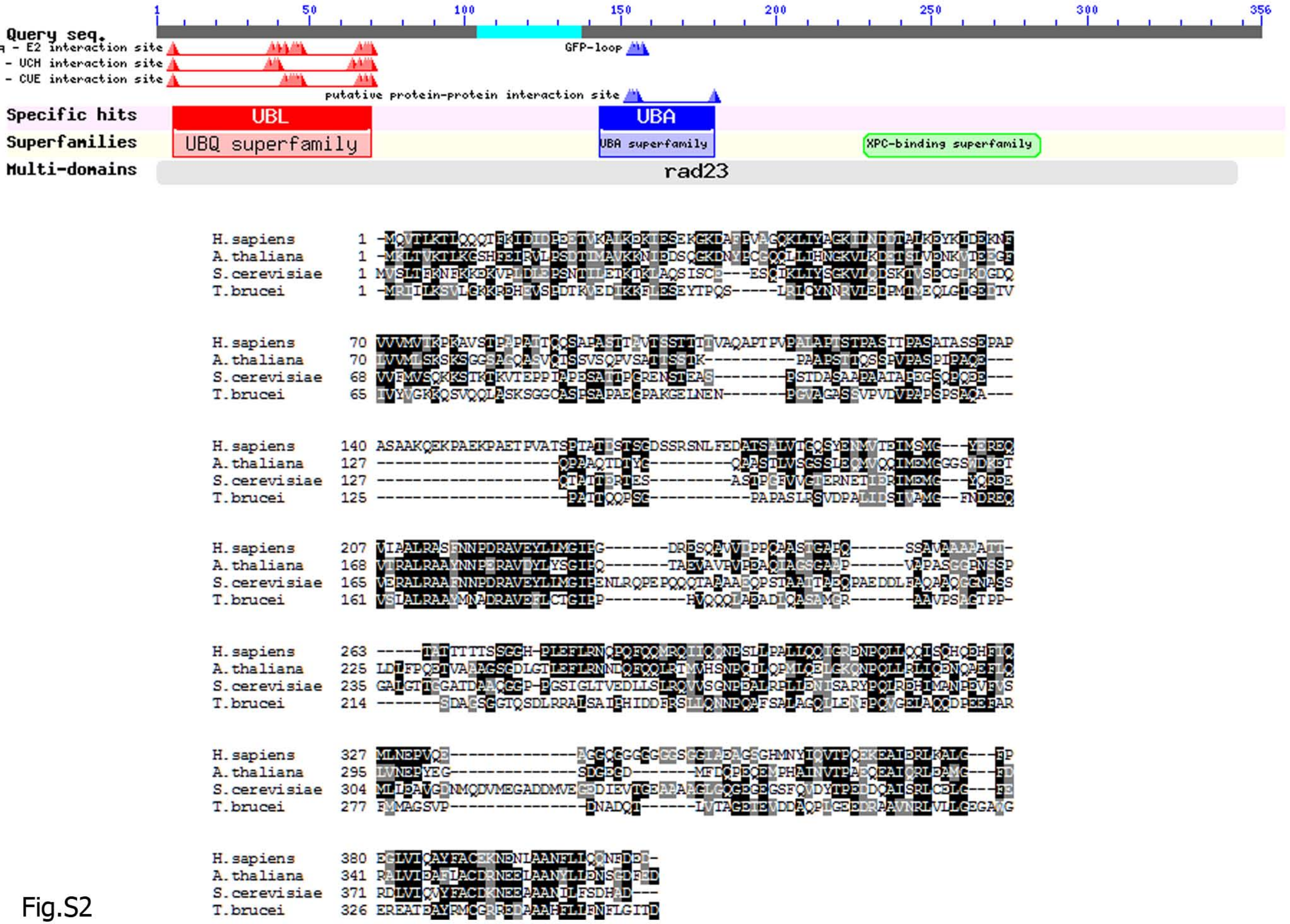

Fig.S2

Fig.S3

T. brucei DDB

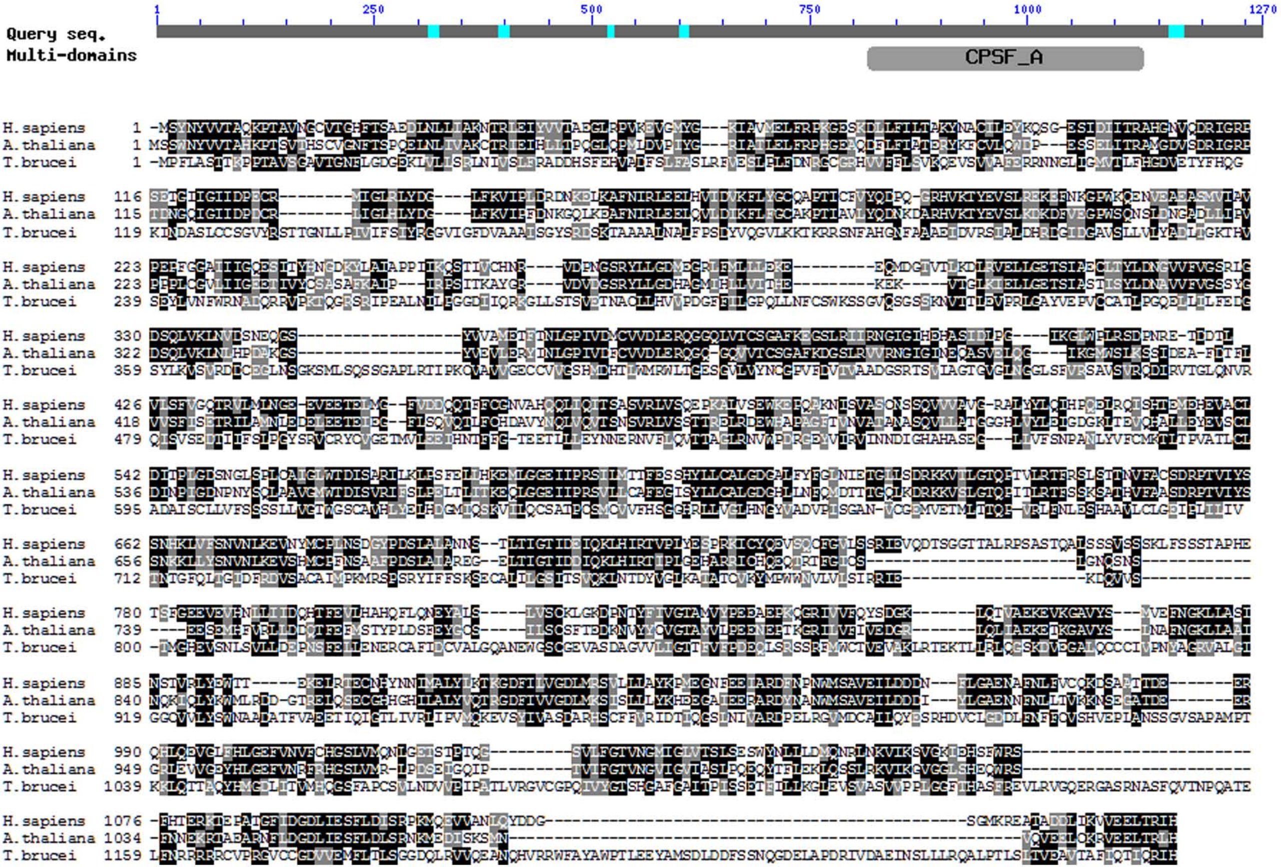

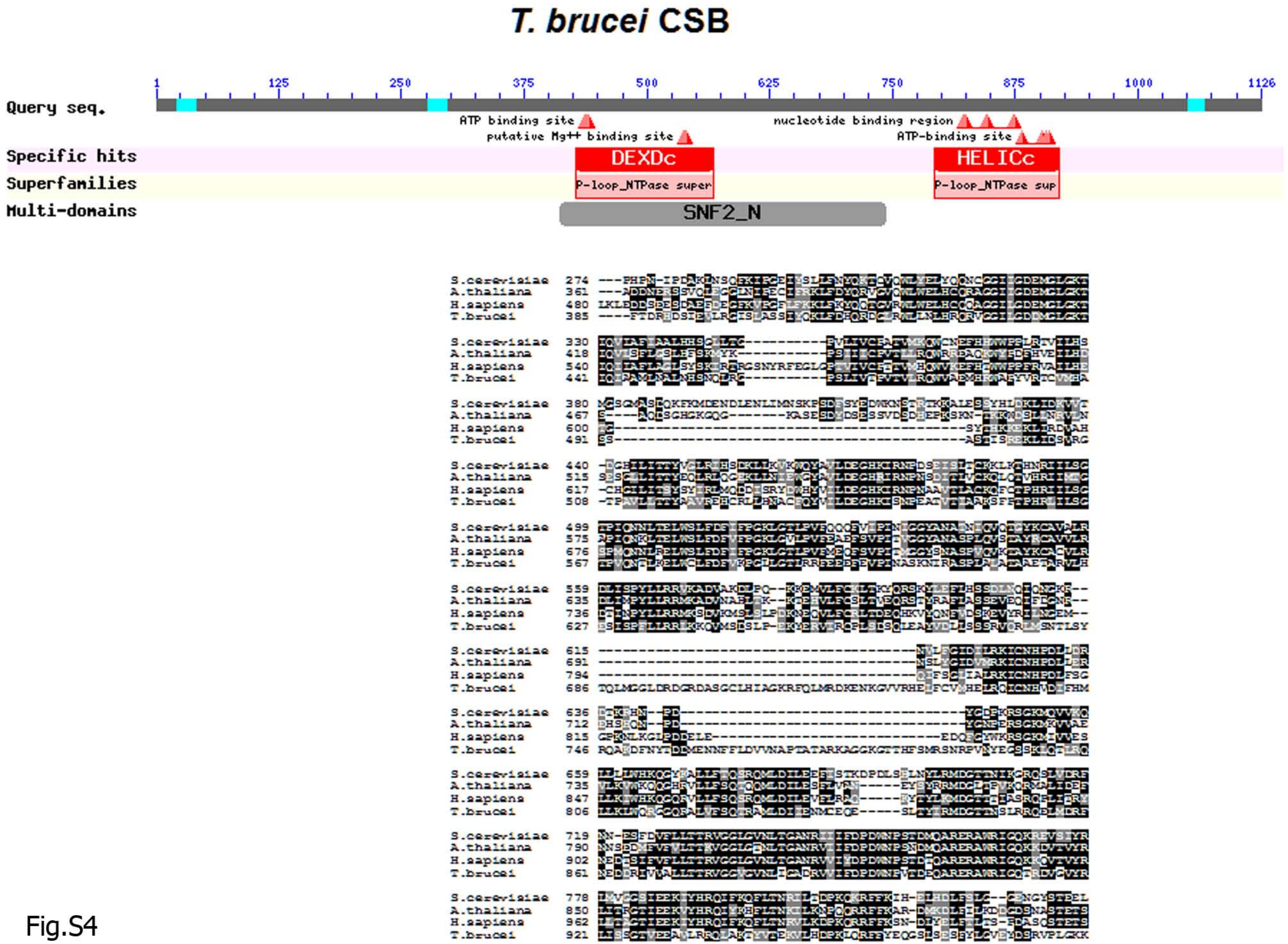
T. brucei CSB
Query seq.
1
125
250
375
500
625
750
875
1000
1126
ATP binding site
putative Mg++ binding site
nucleotide binding region
ATP-binding site
Specific hits
Superfamilies
Multi-domains
DEXDc
HELICc
P-loop_NTPase super
P-loop_NTPase sup
SNF2_N
S.cerevisiae
A.thaliana
H.sapiens
T.brucei

Fig.S4

Fig.S5

T. brucei XPD

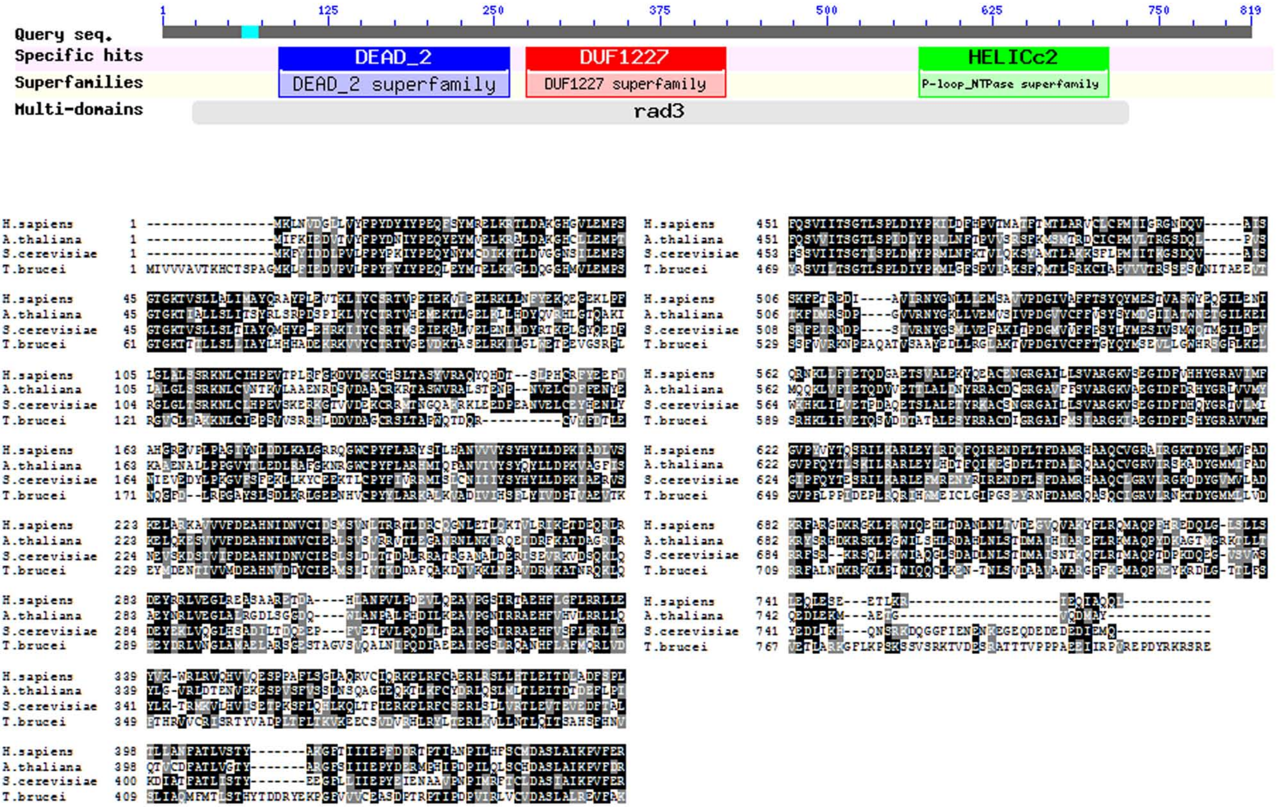

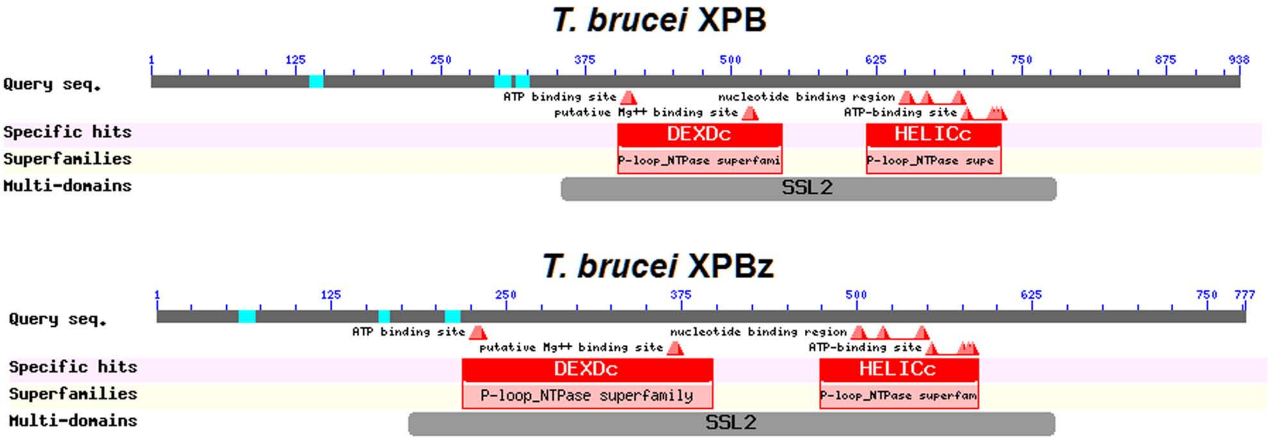
T. brucei XPB
1
125
250
375
500
625
750
875
938
Query seq.
ATP binding site
nucleotide binding region
putative Mg++ binding site
ATP-binding site
Specific hits
DEXDc
HELICc
Superfamilies
P-loop_NTPase superfami
P-loop_NTPase supe
Multi-domains
SSL2
T. brucei XPBz
1
125
250
375
500
625
750
777
Query seq.
ATP binding site
nucleotide binding region
putative Mg++ binding site
ATP-binding site
Specific hits
DEXDc
HELICc
Superfamilies
P-loop_NTPase superfamily
P-loop_NTPase superfam
Multi-domains
SSL2

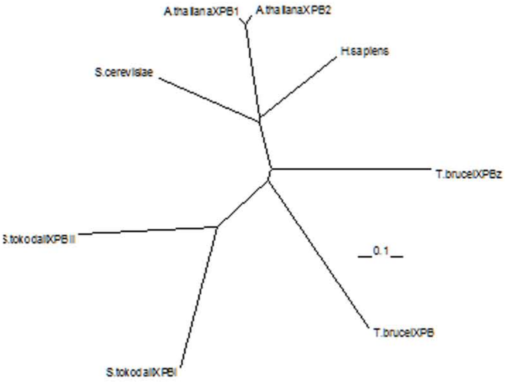
A.thalianaXPB1
A.thalianaXPB2
H.sapiens
S.cerevisiae
T.bruceiXPBz
S.tokodaiiXPBII
0.1
T.bruceiXPB
S.tokodaiiXPBI

Fig.S6

T. brucei XPG

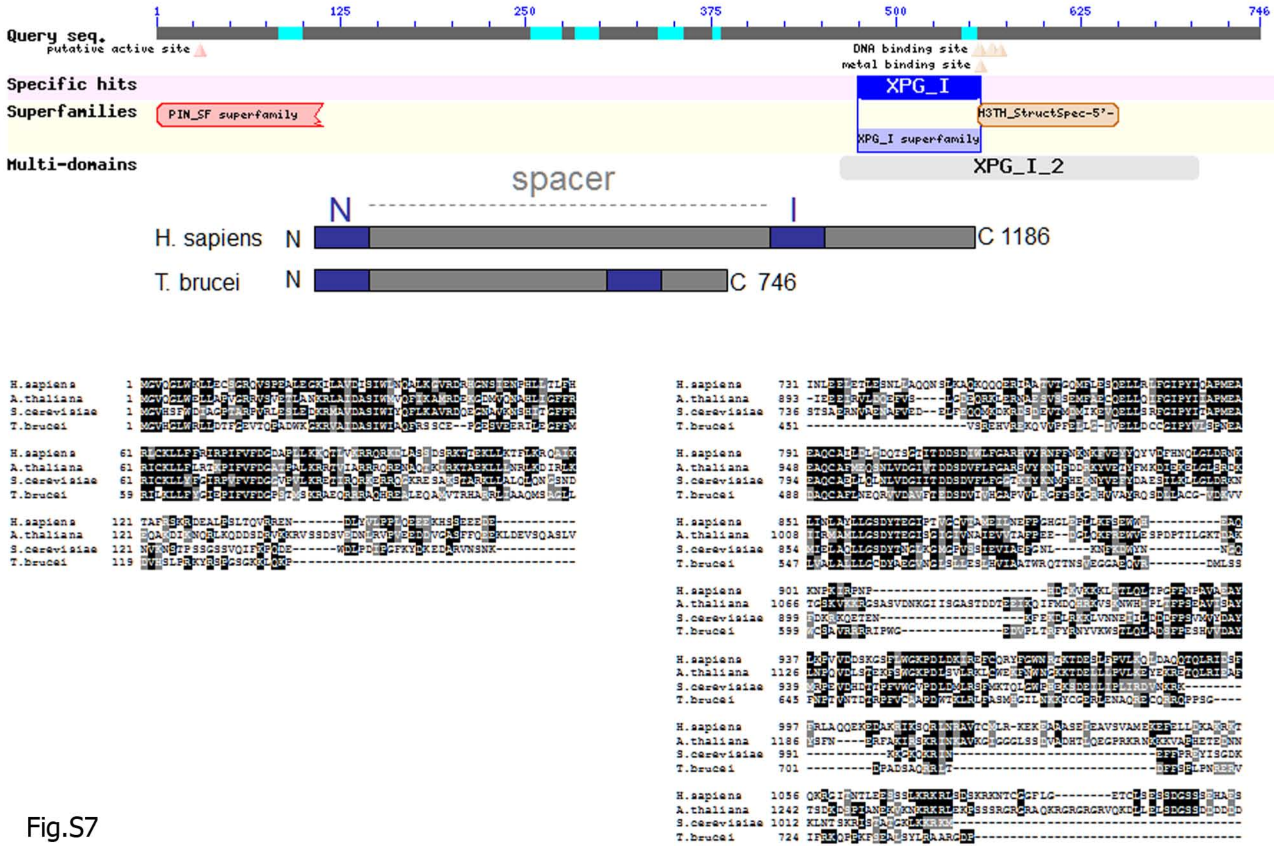

Fig.S7

T. brucei XPF

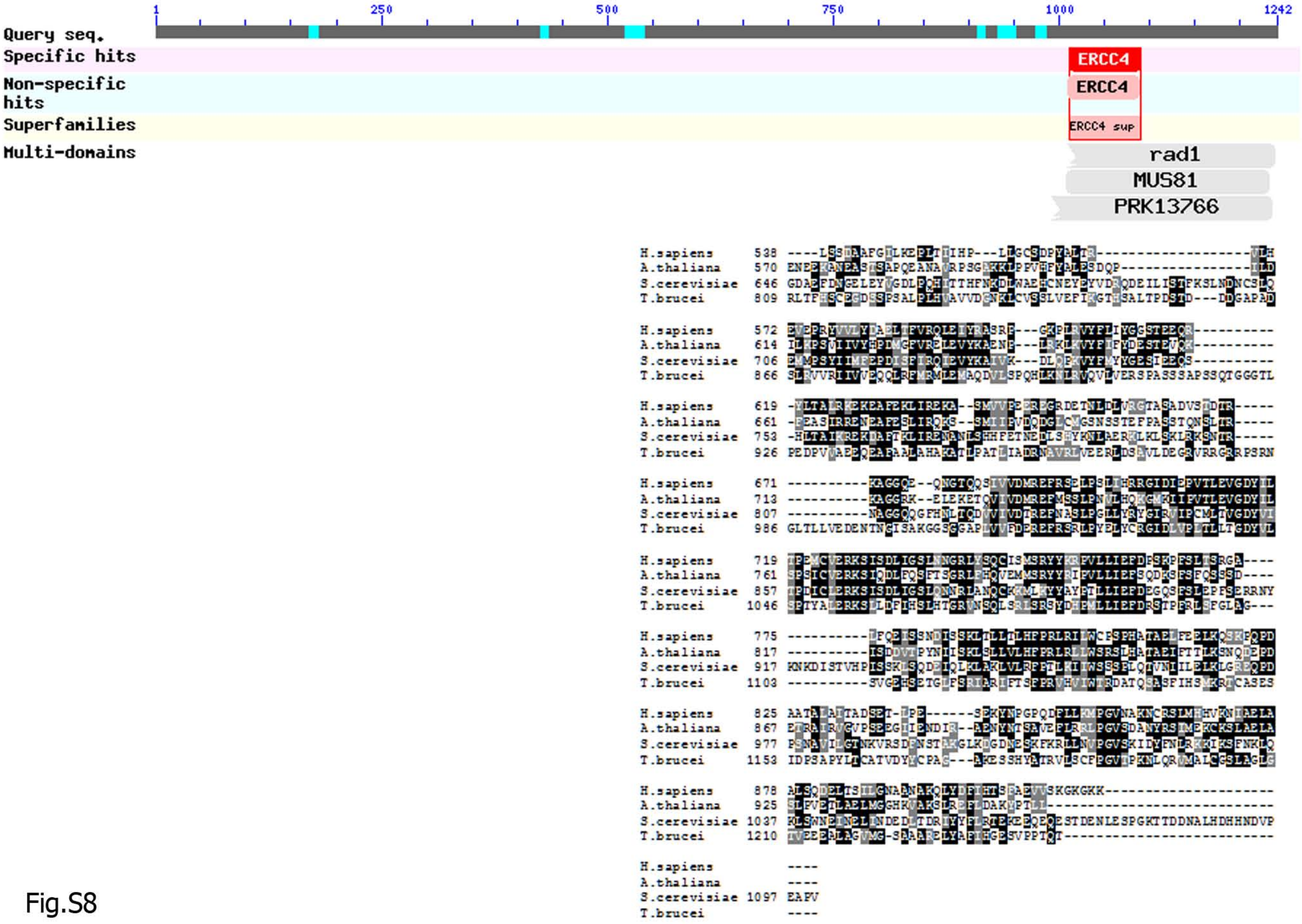

Fig.S8

Fig.S9

T. brucei ERCC1

```
H.sapiens        1 ----MDPGKDKEGVPQPSGPPARKKFVIPLD----------EDEVPPGVAKPLFRSTQSL
A.thaliana       1 MANEDDDGEKSRSLHQQIARKPKTQIVIGVPSYQEVLESSQTKSTPPSLFKPSQSFSQAF
S.cerevisiae     1 --------------MNNTDPTSFESILAGVA------------KIRKEKSGADTTGSQSL
T.brucei         1 ------MPPLPRGVVKVVGALRGDNIIRIMQRHR------YVIEEADDPTYDFLCGGTCV

H.sapiens       47 PTVDTS-AQAAPQTYAEYAISQPLEGAGATCPTGSEPLAGETPNQALKPGAK--------
A.thaliana      61 AFVKSSDVYSPPPPSSAAASSSQPSGASQVPHSSSQTHQTDGASSSSTPVATGSVPSNTT
S.cerevisiae    35 EIDASKLQQQEPQTSRRINSNQVINAFNQQKPEEWTDSKATDDYNRKRPFRS-------T
T.brucei        49 VFVDDADDLSDAARRTKVSQQLSVLKTHTGASWRCVVLLLRVVSEEVRPDIL--------

H.sapiens       98 --SNSIIVSPRQRGNPVLKFVRNVPWEFG-----DVIPDYVLGQSTCALFLSLRYHNLHP
A.thaliana     121 QNRNAILVSHRQKGNPLLKHIRNVKWVFS-----DIIPDYVLGQNSCALYLSLRYHLLHP
S.cerevisiae    88 RPGKTVLVNTTQKENPLLNHLKSTNWRYVSSTGINMIYYDYLVRGRSVLFLTLTYHKLYV
T.brucei       101 --AWLNLHCSVEQGCGVMLFWTDEECAAY------LEGLSDSNVATADYCVGVRRDSTPM

H.sapiens      151 DYIHGRLQSLGKNFALRVLLVQVDVKDPQQALKELAKMCILADCTLILAWSPEEAGRYLE
A.thaliana     176 DYLYFRIRELQKNFKLSVVLCHVDVEDTVKPLLEVTKTALLHDCTLLCAWSMTECARYLE
S.cerevisiae   148 DYISRRMQPLSRNENN-ILIFIVDDNNSEDTLNDITKLCMFNGFTLLLAFNFEQAAKYIE
T.brucei       153 QLLIDALTQTPQLMTRNDVVRAVNSFGSVAGLLTAT----AEQLTELPGFAQKKAGRLHA

H.sapiens      211 TYKAYEQKPADLLMEKLEQDFVSRVTECLTTVKSVNKTDSQTLLTTFGSLEQLIAASRED
A.thaliana     236 TIKVYENKPADLIQGQMDTDYLSRLNHSLTSIRHVNKSDVVTLGSTFGSLAHIIDASMED
S.cerevisiae   207 YLNL--------------------------------------------------------
T.brucei       209 VLNAPFNTSRCLVADVLQRD-QDESNDESSERRPAQETMKQALRCIYDREDEDVQEGGQ-

H.sapiens      271 LALCPGLGPQKARRLFDVLHEPFLKVP---------------------------------
A.thaliana     296 LARCPGIGERKVKRLYDTFHEPFKRATSSYPSVVEPPIPEAPVEKDVNSEEPVEEDEDFV
S.cerevisiae       ------------------------------------------------------------
T.brucei           ------------------------------------------------------------

H.sapiens          ------------------------------------------------------
A.thaliana     356 EDSRKRKKEPEPEKTVKTALSAVFARYSDRLSKKKEKQKEKDTTTASDAETHQN
S.cerevisiae       ------------------------------------------------------
T.brucei           ------------------------------------------------------
```

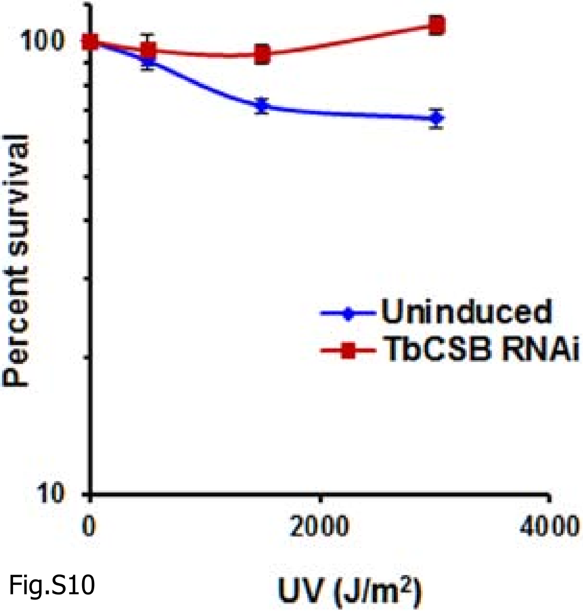
100
10
Percent survival
0
2000
4000
Uninduced
TbCSB RNAi
UV (J/m²)

Fig.S10
